# Impact of Interleukin 10 Deficiency on Intestinal Epithelium Responses to Inflammatory Signals

**DOI:** 10.3389/fimmu.2021.690817

**Published:** 2021-06-16

**Authors:** Stamatia Papoutsopoulou, Liam Pollock, Catherine Walker, William Tench, Sakim Shakh Samad, François Bergey, Luca Lenzi, Raheleh Sheibani-Tezerji, Phillip Rosenstiel, Mohammad Tauqeer Alam, Vitor A. P. Martins Dos Santos, Werner Müller, Barry J. Campbell

**Affiliations:** ^1^ The Henry Wellcome Laboratories of Molecular & Cellular Gastroenterology, Faculty of Health & Life Sciences, University of Liverpool, Liverpool, United Kingdom; ^2^ Department of Biochemistry and Biotechnology, School of Health Sciences, University of Thessaly, Larissa, Greece; ^3^ Lydia Becker Institute of Immunology and Inflammation, Faculty of Biology, Medicine and Health, University of Manchester, Manchester, United Kingdom; ^4^ LifeGlimmer GmbH, Berlin, Germany; ^5^ Centre for Genomic Research (CGR), Department of Evolution, Ecology & Behaviour, University of Liverpool, Liverpool, United Kingdom; ^6^ Institute of Clinical Molecular Biology, Christian Albrechts University of Kiel, Kiel, Germany; ^7^ Warwick Medical School, Bioinformatics Research Technology Platform (RTP), University of Warwick, Coventry, United Kingdom; ^8^ Department of Biology, College of Science, United Arab Emirates University, Al-Ain, United Arab Emirates; ^9^ Laboratory of Systems & Synthetic Biology, Wageningen University & Research, Wageningen, Netherlands

**Keywords:** intestine, enteroids, interleukin 10, tumour necrosis factor, NF*κ*B

## Abstract

Interleukin 10 (IL-10) is a pleiotropic, anti-inflammatory cytokine that has a major protective role in the intestine. Although its production by cells of the innate and adaptive immune system has been extensively studied, its intrinsic role in intestinal epithelial cells is poorly understood. In this study, we utilised both ATAC sequencing and RNA sequencing to define the transcriptional response of murine enteroids to tumour necrosis factor (TNF). We identified that the key early phase drivers of the transcriptional response to TNF within intestinal epithelium were NF*κ*B transcription factor dependent. Using wild-type and *Il10^−/−^* enteroid cultures, we showed an intrinsic, intestinal epithelium specific effect of IL-10 deficiency on TNF-induced gene transcription, with significant downregulation of identified NF*κ*B target genes *Tnf*, *Ccl20*, and *Cxcl10*, and delayed overexpression of NF*κ*B inhibitor encoding genes, *Nfkbia* and *Tnfaip3*. IL-10 deficiency, or immunoblockade of IL-10 receptor, impacted on TNF-induced endogenous NF*κ*B activity and downstream NF*κ*B target gene transcription. Intestinal epithelium-derived IL-10 appears to play a crucial role as a positive regulator of the canonical NF*κ*B pathway, contributing to maintenance of intestinal homeostasis. This is particularly important in the context of an inflammatory environment and highlights the potential for future tissue-targeted IL-10 therapeutic intervention.

## Introduction

Interleukin 10 (IL-10) is an anti-inflammatory cytokine that is synthesized and secreted by lymphocytes (T and B cells), macrophages, dendritic cells, and some epithelial cells (1, 2). Its anti-inflammatory role has been studied extensively, including its protective effect in innate and adaptive immune responses (3), the central nervous system (4), in tissue fibrosis (5), and atherosclerosis (6). The major role of IL-10 in intestinal homeostasis was revealed by genetic intervention in mice, where it was shown that both IL-10 deficient and IL-10 receptor deficient mice develop spontaneous colitis (7, 8). Also, CD1d deficiency has been shown to affect *Il10* transcription in intestinal epithelium, which was linked to increased mortality in an oxazolone-induced murine colitis model (9). In humans, defective IL-10/IL-10R signalling has been shown to correlate with inflammatory bowel disease (IBD) (10, 11). Genome-wide association studies have identified polymorphisms in *IL10* and *IL10R* genes, both associated with early onset of IBD, more severe disease, and lack of response to standard treatments, including anti-tumour necrosis factor (TNF) biologic therapy (12, 13). Further studies using transgenic animals have revealed that IL-10 receptor signalling in macrophages (14, 15) and T cells (16) is critical for intestinal homeostasis and that absence contributes to the development of intestinal inflammation. Moreover, murine intestinal epithelial cells express functional IL-10 receptor, that upon addition of IL-10 downregulate cell-surface major histocompatibility complex (MHC) class II molecule expression induced by interferon gamma (17).

IL-10 can exert its function as an anti-inflammatory cytokine through activation of the Janus kinases/signal transducer and activator of transcription proteins (JAK/STAT) pathway (18). Importantly however, IL-10 also affects the nuclear factor kappa B (NF*κ*B) transcription factor pathway, a key signalling pathway essential for mediation of inflammation in response to tissue damage and infection (19). The NF*κ*B family of transcription factors includes five subunits, p65 or RelA, p50, c-Rel, p52, and RelB, that may function as homo- or hetero-dimers (20). These dimers are retained in an inactive state in the cytoplasm by binding to a member of the family of inhibitory *κ*B proteins (I*κ*Bs). Upon stimulation, I*κ*Bs are degraded, and the NF*κ*B active dimers translocate to the nucleus to regulate transcription. NF*κ*B activation is essential for intestinal epithelium homeostasis, maintaining barrier integrity, through regulation of cellular proliferation, differentiation, and survival, and mediating signalling and interaction between the mucosal immune system and resident microbiota (21). Severe chronic intestinal inflammation in mice can result from intestinal epithelial-specific ablation of I*κ*B kinases (IKKs), with NF*κ*B deficiency also leading to enhanced apoptosis of colonocytes, impaired levels of antimicrobial peptides, and enhanced translocation of bacteria across the mucosa (22). Important in immune response and tolerance, it has been shown recently that NF*κ*B activation is necessary for differentiation of M-cells of the follicle-associated epithelium overlying Peyer’s patches (23). In monocytes, IL-10 can block NF*κB* activation either through inhibiting the canonical pathway (p50/p65) (24) or inducing the nuclear translocation of the inhibitory homodimer p50/p50 (25). To date there are no studies evaluating the impact of epithelial cell derived IL-10 on NF*κ*B signalling dynamics in the intestinal epithelium.

In this study, we applied both Assay for Transposase-Accessible Chromatin (ATAC) sequencing and RNA sequencing to murine enteroids so as to understand the transcriptional profile response to stimulation with TNF, a key pro-inflammatory cytokine found at high levels in the inflamed intestinal mucosa of both mouse (26) and human (27). We validated identified target genes by qPCR in enteroids stimulated with TNF, or with another key pro-inflammatory stimulus of intestinal epithelium, bacterial flagellin (28). qPCR analysis revealed for the first time that IL-10 deficient enteroids showed a differential response to inflammatory stimuli, affecting known targets of the canonical NF*κ*B pathway. These results were further validated by dynamically measuring endogenous NF*κ*B activation, using a lentivirus-based reporter assay and immunoblockade approaches in TNF-stimulated enteroid cultures.

## Materials and Methods

### Mice

Interleukin 10 knockout (*Il10^−/−^*) mutant mice (B6.129P2-Il10^tm1Cgn^/J), backcrossed to the C57BL/6J genetic background for several generations, were obtained from The Jackson Laboratory (Bar Harbor ME, USA); Stock No. 002251; www.jax.org/strain/002251) and imported *via* Charles River UK Ltd (Margate, UK). Matched C57BL/6J substrain control mice were provided by Charles River Ltd. Mice were bred and co-housed in a specific-pathogen-free environment at the Biomedical Services Unit, University of Liverpool. Mice were culled by rising CO_2_, followed with cervical dislocation. Proximal small intestine was dissected and transported on ice in sterile phosphate-buffered saline (PBS) pH 7.4, for use in establishing enteroid cultures.

#### Ethics Statement

All animal experiments were performed in accordance with regulations of the Home Office of the United Kingdom under Home Office Licence (PPL: 70/8457).

### Enteroid Cultures

After dissection, 1–2 cm sections of C57BL/6J or *Il10^−/−^* proximal small intestine were cut open longitudinally and divided further into smaller sections. Sections were flushed with ice-cold sterile PBS pH 7.3, approximately 10 times until clear. Tissue sections were processed in 2 mM ethylenediaminetetraacetic acid (EDTA) chelation buffer to generate intestinal crypts, which were then resuspended in Matrigel (Corning; Loughborough, UK) and cultured over 7 days to generate enteroids as previously described (29). Cultures were passaged at day 7 by mechanically disruption through a 27G needle, resuspended in Matrigel, and plated out again on 24-well plates. Experimental treatment of enteroid cultures was only performed following a minimum of one passage. Cryo-stored intestinal crypts from hTNF.LucBAC mice, that express luciferase under the control of the human TNF promoter (30), were used to generate enteroids to study IL-10 receptor (IL-10R) signalling. For IL-10R immunoblockade experiments, enteroids were pre-treated overnight using 1 μg/ml purified anti-mouse CD210 (IL-10R) antibody (Clone 1B1.3a) and compared to enteroid cultures pre-treated with rat IgG1, κ isotype control antibody (Clone RTK2071) (BioLegend UK Ltd; London, UK). For *in vitro* stimulation, enteroid cultures were left untreated or stimulated with 40 ng/ml mouse recombinant TNF (Peprotech Ltd., London, UK) or 100 ng/ml flagellin (Invivogen; Toulouse, France), at doses known to stimulate intestinal epithelial cells (28).

### Assay for Transposase-Accessible Chromatin Sequencing

The ATAC sequencing protocol was based on the protocol of Buenrostro et al. (31) and modified for the specific cell type, as described below. Enteroid cultures were maintained in 24-well plates, as described above, and they were either left unstimulated or they were treated with 40 ng/ml TNF for 2 h (four wells per condition). At the end of stimulation, the medium was removed, the plate was transferred on ice, and 1 ml cold PBS was added in each well. Organoids were resuspended, and the four wells were pooled into one 15 ml Falcon tube that already contained 10 ml of cold PBS. Cells were precipitated by centrifugation at 500 × *g* for 10 min, at 4°C. The cell pellet was resuspended in 200 μl 1× Trypsin-EDTA solution (Sigma) and incubated for 3 min, at 37°C. The reaction was terminated by addition of 10 ml cold PBS containing 1% (w/v) bovine serum albumin (BSA; Sigma). Cells were precipitated by centrifugation (500 × *g* for 10 min, at 4°C), and the cell pellet was resuspended in 500 μl sterile PBS. Then 50,000 cells were aliquoted in 1.5 ml Eppendorf tubes, washed once with 1 ml cold PBS, followed by another wash with 1 ml of resuspension buffer (10 mM Tris/HCl pH 7.5, 10 mM NaCl, 3 mM MgCl_2_). The cell pellet was resuspended in 100 μl freshly made lysis buffer (resuspension buffer containing 0.1% (v/v) Nonidet P-40 and 0.1% (v/v) Tween-20) for 3 min on ice. The reaction was stopped by addition of 1 ml cold wash buffer [resuspension buffer containing 0.1% (v/v) Tween-20], and the nuclei were pelleted by centrifugation (1,000 × *g* for 10 min, at 4°C). The pellet was resuspended in 50 μl transposition reaction mix (32), composing of 25 μl 2× TD Buffer (Illumina; Little Chesterford, UK), 16.5 μl 1× PBS, 0.1% (v/v) Tween-20, 0.01% v/v Digitonin (Promega; Southampton, UK), 1.2 μl TDE1 Tagment DNA enzyme (Illumina) and incubated for 10 min on thermomixer (1,000 rpm, at 37°C). At the end of the incubation, the genomic DNA was purified with MinElute Reaction Cleanup Kit (Qiagen; Manchester, UK), eluted in 10 μl BE buffer and stored at −20°C until further use. The tagmented DNA (10 μl) was amplified by PCR to generate the library in a 50 μl reaction mix containing 25 μl NEBNext High-Fidelity 2×PCR Master Mix, 2.5 μl forward primer Ad1.2_502 (25 μM, 5′-aatgatacggcgaccaccgagatctacacctctctattcgtcggcagcgtc-3′) and 2.5 μl reverse indexing primer Ad2.5_705 (25 μM, 5′-caagcagaagacggcatacgagataggagtccgtctcgtgggctcggagatgt-3′). The PCR reactions were carried out on a qPCR LightCycler 480 (Roche; Basel, Switzerland). The PCR conditions were: 1 cycle of 72°C/5 min, 15 cycles of 98°C/30 s, 98°C/10 s, 63°C/30 s, and one cycle of 72°C/1 min. The library was purified by Agencourt AMPure XP magnetic beads (Beckman Coulter; High Wycombe, UK), and 1 μl of each library was assessed for quality by high sensitivity DNA assay. Samples were analysed on the Illumina NovaSeq using SP chemistry (Paired-end, 2 × 150 bp sequencing, generating an estimated 325 million clusters per lane). The paired-end sequencing data obtained from the analysis for the assay for transposase accessible chromatin experiment, were post-processed to remove the sequencing adapters using (33). The resulting sequences were then aligned onto the mouse genome version mm10.3.0.0, by using bowtie2 (version 2.3.4.3) (34) with the following options ‘—very-sensitive’ and ‘—k 10’. The peak identification on the alignment result were obtained using the ‘Genrich’ software with the options ‘-j’ ‘-y’ ‘-r’, ‘-e MT’. The annotation for the identified peaks and the comparison between samples, were performed by a custom R script using the Ensembl genome annotation (August 2020). Bed and Bigwig format files were uploaded for visualisation purposes on integrative genomics viewer (IGV) application (35).

### RNA Sequencing

Host transcriptome analysis was performed by RNA sequencing of unstimulated and TNF (40 ng/ml) stimulated enteroid cultures from C57BL/6J mice (N = 3). RNA extraction and purification from enteroids were performed using the RNeasy mini kit (Qiagen), as per manufacturer’s instructions. Strand-specific sequencing libraries were prepared with the TruSeq stranded Total RNA kit (Illumina) from 1 µg total RNA of each sample and sequenced on an Illumina HiSeq2000 (100-nucleotide paired-end reads).

#### Read Mapping and Analysis of Differential Expression

Raw reads passing the chastity filter from Illumina were first pre-processed using cutadapt (36) and PrinSeq-lite (37) to remove adapter and low quality sequences. The reads were aligned to non-repeat masked version of the *Mus. musculus* reference genome (GRCm38) using TopHat2 (38), while the corresponding GTF annotation file was obtained from the Ensembl database (Mus_musculus.GRCm38.80.gtf). The DESeq2 package from BioConductor was used for differential expression analysis (39). DESeq2 is based on a model using the negative binomial distribution and uses a normalization procedure based on sequencing depth and biological variance. Log2 fold changes (between stimulated and unstimulated control) and adjusted p-values (corrected for multiple testing using the Benjamini and Hochberg method) were calculated, and genes were selected as differentially expressed with an absolute fold-change ≥2 and adjusted *p*-value of <0.05.

#### Cluster and Pathway Analysis

Hierarchical cluster analysis of RNA sequencing datasets was performed using Cluster 3.0 (http://bonsai.hgc.jp/~mdehoon/software/cluster/software.htm) (40) with nodes and correlations identified using Java Treeview v1.59 (http://jtreeview.sourceforge.net) (41). Identification of NF*κ*B target genes within the sequencing datasets was supported using gene resources available at The Gilmore Laboratory, Cell & Molecular Biology Division, Biology Department, Boston University, Boston MA, USA (http://www.bu.edu/nf-kb/gene-resources/target-genes/) and additional database/publication resources (42–45). Gene ontology enrichment analysis was performed based on the Panther classification system that was designed to classify proteins (and their genes) in order to facilitate high-throughput analysis [GO enrichment analysis (geneontology.org)] (46, 47). In order to establish whether the TNF-induced immediate–early response genes identified by RNA-sequencing share any common transcription factors, we utilised the freely available PASTAA tool at http://trap.molgen.mpg.de (48). This allowed us to predict affinities of transcription factors to promoters of those genes. A cut-off was applied at *p <* 0.05 for obtained hypergeometric *P*-values, and this was used to generate a final list of ranked predictions.

### RNA Extraction and qPCR

RNA extraction and purification from enteroids were performed on samples cryostored in RLT buffer using the RNeasy mini kit (Qiagen), as per manufacturer’s instructions. Tissues isolated from mice following intraperitoneal injection of TNF were disrupted in RLT buffer using bead-beating in a TissueLyser II (Qiagen), and total RNA was purified using the RNeasy mini kit. For all samples, purified RNA was reverse transcribed using the High-Capacity RNA-to-cDNA Kit (Applied Biosystems; Paisley, UK), and cDNA stored at −20°C. Reactions for real time PCR (qPCR) were performed using 96-well plates, with full reaction mix consisting of Taqman Fast advanced master mix (Applied Biosystems), Taqman Gene Expression Assay probes (Applied Biosystems), and 50 ng total cDNA as per manufacturer’s instructions. All reactions were carried out on a qPCR LightCycler 480 (Roche). Conditions for qPCR were as follows: one cycle of: 120 s at 50°C, 20 s at 95°C; 40 cycles of 3 s at 95°C, 30 s at 60°C, and 20 s at 60°C; one cycle at 120 s at 72°C and 30 s at 60°C. Cp values were calculated from 2^nd^ derivative analysis and relative quantification was calculated using 2-ΔΔCT method (49). Gene expression probes were *Tnf (*Mm00443258_m1), *Tnfaip3 (*Mm00437121_m1), *Nfkbia (*Mm00477798_m1), *Il10* (Mm01288386_m1), *Il10ra* (Mm00434151_m1), *Tnip1* (Mm01288484_m1), *Tnip2* (Mm00460482_m1), *Irf1* (Mm01288580_m1), *Nfkbiz* (Mm00600522_m1), *Ccl20* (Mm01268754_m1), and *Cxcl10* (Mm00445235_m1). All results were normalized to the expression of housekeeping gene *Gapdh* (Mm99999915_g1).

### Lentiviral Transduction of Enteroids for Assay of NF*κ*B Activation

NF*κ*B transcriptional activity was monitored using a lentiviral construct (*κ*B-NLSluc) that expresses firefly luciferase under the control of the classical NF*κ*B promoter. This construct is a reporter for NF*κ*B (5*κ*B-Luc) in which the 5× repeat *κ*B consensus sequence was introduced into the luciferase vector, constructed, produce, and used as previously described (50–52). For lentivirus transduction, the enteroid/Matrigel mixture was solubilised in 1 ml ice-cold PBS and further diluted with 9 ml ice-cold PBS, and the sample was centrifuged at 600 × *g* for 5 min. The cell pellet was resuspended in 0.2 ml 1× trypsin and the sample was incubated for 3 min at 37°C. Trypsinisation was terminated by addition of 10 ml PBS, and the cells were pelleted by centrifugation at 600 × *g* for 5 min. The cells were resuspended 0.1 ml Intesticult containing 10 μM Y27632 (Sigma), and equal volume of virus was added. TransduxTM reagent was added (200×; SBI System Biosciences; Palo Alto CA, USA), and the mixture was spinoculated for 30 min at 800 × *g* at 4°C. Afterwards, the cell suspension was plated on a layer Matrigel (2D culture) and left to grow for 72 h, with one change of medium at 24 h post-transduction. On the day of stimulation, D-luciferine (Biosynth AG; Staad, Switzerland) was added to the medium, to a final concentration of 1 mM, and cultures were incubated for 20 min, followed by addition of 100 ng/ml TNF for 3 h. At the end of stimulation, the cells were treated with 0.2 ml gentle cell dissociation buffer (STEMCELL Technologies UK Ltd), and luminescence was measured in luminometer (FB12 Single Tube Luminometer (Titertek-Berthold; Harpenden, UK).

### Assay of Secreted IL-10 Protein From Murine Enteroid Cultures

Enzyme linked immunosorbent assay (ELISA) was used to measure secreted IL-10 protein levels from murine enteroids treated with 40 ng/ml TNF over 8 h, as per manufacturer’s instructions (R&D Systems; Abingdon, UK).

### Statistical Analysis of *In Vitro* and *In Vivo* Experimental Datasets

Results were expressed as mean ± standard error of mean (SEM). Statistical inferences on data were performed using non-parametric Kruskal–Wallis test, followed by all pairwise comparisons of all treatment means (StatsDirect v3.0.171—StatsDirect Ltd; Birkenhead, UK). Differences were considered statistically significant when *p <*0.05.

## Results

### ATAC Sequencing of Enteroids

**Figure 1:**
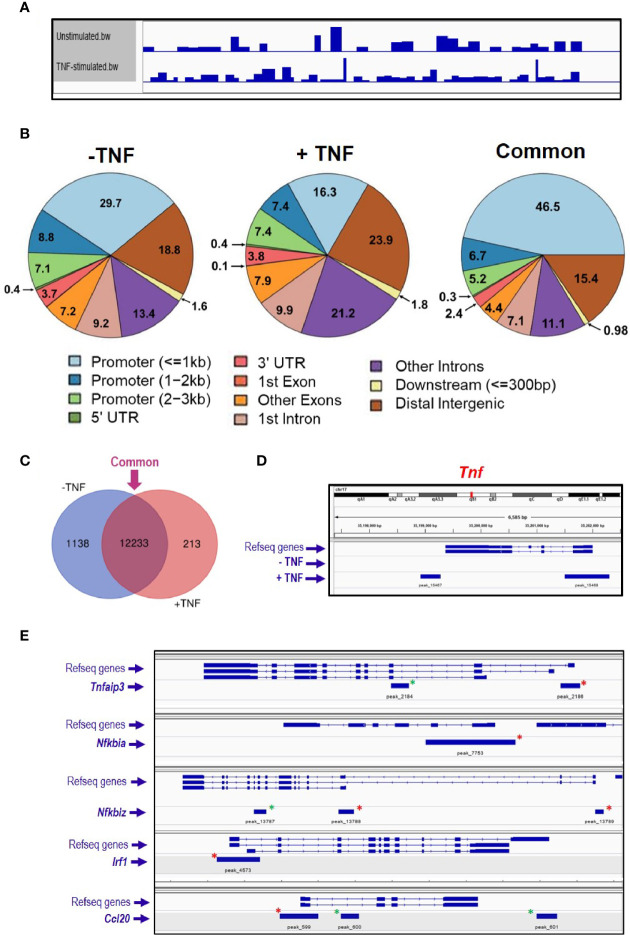
ATAC sequencing analysis of TNF-stimulated enteroids. Enteroid cultures were left unstimulated or were stimulated with 40 ng/ml TNF for 2 h. DNA isolated from these cultures was further processed, amplified by PCR, and DNA sequencing followed by informatics analysis. **(A)** Genome coverage in both experimental conditions analysed, and **(B)** charts illustrating the percentage of annotated peaks identified within different genomic regions, with percentages shown to one decimal place. Informatics analysis grouped the peaks into three groups; 1) unstimulated only (− TNF), 2) TNF-induced only (+ TNF) and 3) ‘common’, which refers to peaks in both the unstimulated and stimulated sample. **(C)** Venn diagram comparing the unstimulated and stimulated samples, illustrating the number of identified genes as having open chromatin in their promotor. Venn diagram software from Ghent University, freely available at http://bioinformatics.psb.ugent.be/webtools/Venn/. **(D)** *Tnf* locus and peak annotation in unstimulated and stimulated conditions. **(E)**. Five classical NF*κ*B signaling pathway genes identified by ATAC-sequencing as having open chromatin loci. Green asterisks indicate annotated peaks unique to the stimulated sample. Red asterisks highlight the peaks ‘common’ to both conditions.

The ATAC sequencing protocol has been developed for mouse enteroids aiming to identify genome-wide chromatin accessible regions, which are known to reflect active transcription (53). Untreated enteroids were trypsinised into single cell suspension, and 50,000 cells were used for consequent steps, as less cells (5,000 or 10,000) did not give adequate DNA yield to create a library. The tagmentation reaction is critical for the DNA fragment size, because an ideal library size ranges from 200 bp to 1 kb. Therefore, different incubation times were tested, 10, 20, 30, 60, 75, and 90 min, and the DNA fragments were accessed on a DNA bioanalyzer. Based on data obtained we decided to perform the tagmentation reaction for 10 min at 37°C (**Supplementary Information—Figure S1**). Moreover, the number of PCR cycles needed for the DNA library synthesis has to be experimentally calculated. For this purpose, real time qPCR analysis was performed and showed that 15 cycles were sufficient to amplify the tagmented DNA without reaching the plateau phase (**Supplementary Information—Figure S1**). Small intestinal organoid cultures were either left untreated, or they were stimulated with 40 ng/ml TNF for 2 h (N = 1 mouse per group). At the end of the stimulation all cultures were processed simultaneously as described in ‘*Materials and Methods*’, and sequencing analysis was performed in the final DNA libraries. Coverage of the mouse genome within the samples was visualised by IGV viewer (**Figure 1A**). Informatics analysis grouped the identified peaks into three groups; 1) unstimulated only, 2) TNF-induced only and 3) ‘common’, which refers to peaks in both the unstimulated and stimulated sample (**Figure 1B**). Further comparison revealed that the majority of the identified genes have open chromatin in their promoter (up to 3 kb from their transcription start site) belonged to the ‘common’ group (**Figure 1C**). In the TNF-induced sample, the total peak number was 2,954 with 918 (~31%) peaks located in the promoter region, the known regulatory DNA element of gene transcription. Within the promoter, 52% of the peaks appeared in less than 1 kb from the transcription initiation site. Amongst those genes identified, *Tnf* (encoding TNF) appeared to have open chromatin within its promotor region (**Figure 1D**). The genes linked to those 918 peaks were subsequently submitted for gene ontology enrichment analysis, which identified GTPase signalling pathways among the most significant pathways in enteroids upon TNF treatment (**Supplementary Information—Table S1**). In the ‘common’ group, we identified some known genes involved in classical NF*κ*B signalling pathway, such as *Nfkbia* (encoding I*κ*Bα, the prototype inhibitor of NF*κ*B), *Tnfaip3* [encoding A20, an inducible inhibitor of NF*κ*B (54, 55)], *Nfkbiz* [encoding a member of the ankyrin-repeat family with high sequence similarity to the I*κ*B family of proteins (56)], and NF*κ*B target genes such as *Irf1* [encoding an activator of interferons (57)], and *Ccl20* [encoding a chemokine recruited to inflammatory sites (58)]; see **Figure 1E**.

### RNA Sequencing of Enteroids

**Figure 2:**
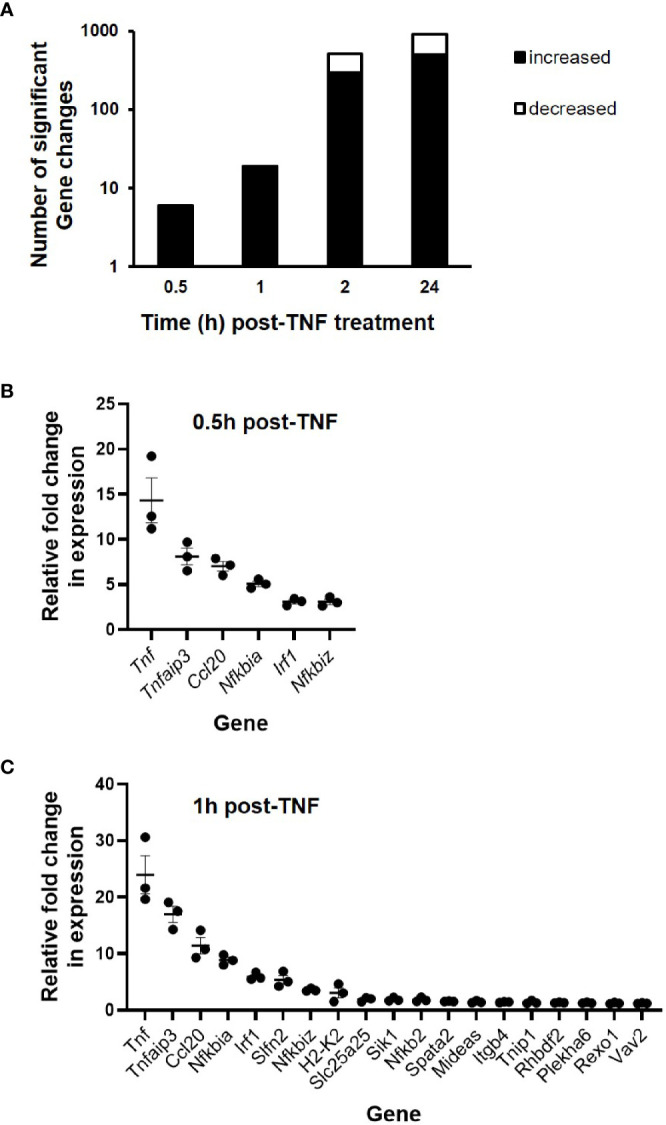
Gene expression changes in TNF-stimulated C57BL/6J enteroids as assessed by RNA sequencing. Enteroid cultures were left unstimulated or stimulated with 40 ng/ml TNF for 0.5, 1, 2, and 24 h. Total RNA was isolated, sequencing was performed, followed by informatics analysis. **(A)** Total number of differentially expressed genes across all time points sampled and fold change in expression of specific genes at **(B)** 0.5, and **(C)** 1 h post-TNF treatment (N = 3 mice). All genes presented were identified as being significantly upregulated; *p* < 0.05 (calculated based on log2 fold changes between stimulated and unstimulated controls, and corrected for multiple testing using the Benjamini and Hochberg method).

To further examine the transcriptional response of intestinal epithelium to inflammatory environment, C57BL/6J enteroids (N = 3 mice per group) were left untreated or were stimulated with 40 ng/ml TNF for a time course up to 24 h. RNA sequencing analysis revealed 1,141 genes that showed statistically significant change in expression compared to untreated controls (*p* < 0.05) (**Figure 2A**). Analysis of the early phase response, during the first hour, showed only six genes significantly upregulated at 0.5 h post treatment and 19 genes upregulated at 1 h, whereas no genes were observed to be downregulated during the first hour of stimulation. At 2 h, 296 genes were significantly elevated, with a further 213 genes identified to be significantly downregulated. Late phase response to TNF induction saw 503 genes significantly increased in their expression, and 412 genes observed to be downregulated. Of the 1,141 genes identified, 213 showed ± two-fold change in expression at one or more time points (0.5, 1, 2, and/or 24 h) compared to unstimulated control enteroids, and our study was further focused on them. *Tnf* was the most upregulated gene in the first two time points, showing 15- and 27-fold changes, closely followed by *Tnfaip3* that showed nine- and twenty-fold upregulation (**Figures 2B, C**). Overall, all six genes 0.5 h post-TNF treatment showed significant upregulation at 1 h as well.

**Figure 3:**
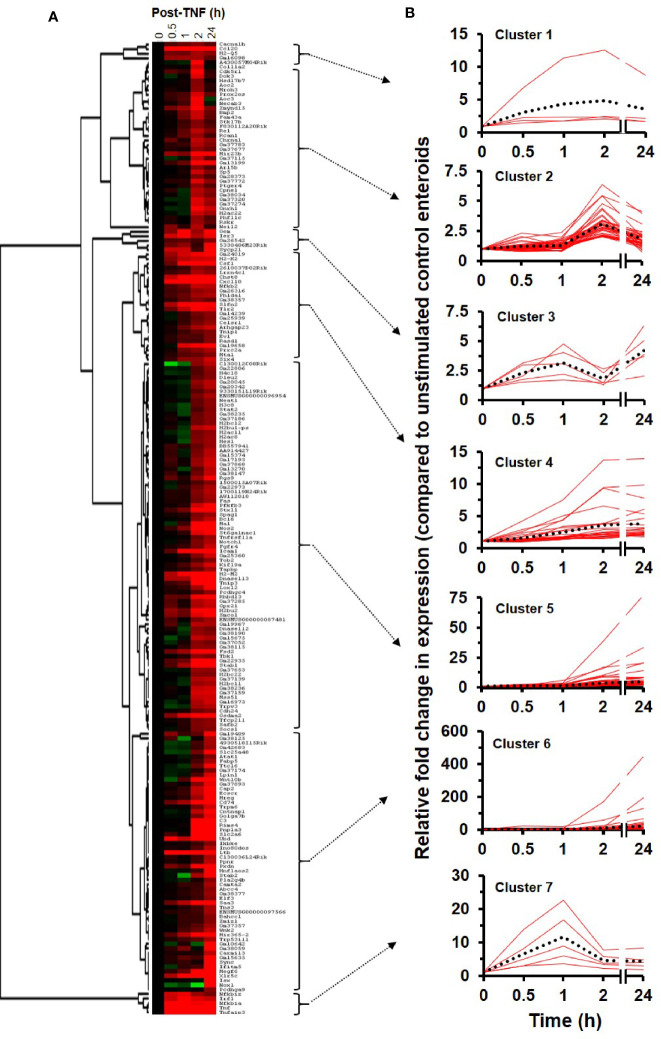
RNA sequencing cluster analysis of TNF-induced enteroids. **(A)** Heatmap and dendrogram of significantly altered genes (± two-fold change) at time points 0.5, 1, 2, and 24 h post-treatment with 40 ng/ml TNF compared to unstimulated controls. **(B)** Cluster correlation analysis identified seven gene groups (Clusters 1–7), with each graph illustrating the dynamic expression profile of individual genes compared to unstimulated controls (0 h). Genes (red lines) are plotted alongside the trend-line (dotted black line) for that cluster so that conformity to the trend can be observed. Data of the relative fold changes to controls for each gene within the clusters are provided in **Supplementary Information—Table S2**.

Following hierarchical heatmap cluster analysis, seven distinct groups of genes were identified, see **Figure 3A**. In Cluster 1, genes (n = 4) showed a pattern of expression increasing above unstimulated control levels from 0.5 h, peaking at 2 h post-TNF stimulation, before decreasing in expression by 24 h; Cluster 2 genes (n = 37) showed little increase in expression up to 1 h, with peak only at 2 h before lowering in levels at 24 h. For Cluster 3, genes (n = 5) showed a biphasic response, peaking initially at 1 h post TNF stimulation, falling back to basal at 2 h, and then seen to be elevated in expression again at 24 h. Cluster 4 contained genes (n = 24) that displayed a steady increase in expression throughout the time-period, with the majority plateauing at 24 h. Cluster 5 genes (n = 81) showed relatively little change in the first hour, followed by a consistent increase in expression from 2 to 24 h. Similarly, Cluster 6 genes (n = 57) showed a similar pattern to Cluster 5, but with some genes dramatically elevated in expression only at 2 h continuing through to higher levels at 24 h. Cluster 7 genes (n = 5) showed early phase increased in expression from 0.5 h peaking at 1 h, before dropping rapidly towards basal levels by 2 h; see **Figure 3B**. Data for each gene, of their relative fold changes to controls within the identified clusters, are provided in **Supplementary Information—Table S2**.

A comparison was made between TNF-induced genes identified as significantly upregulated (± two-fold) by RNA sequencing (n = 296, at 2 h) and those ATAC sequencing genes with annotated peaks within the promoter region. Comparison of the RNAseq dataset with peaks identified in the ATACseq ‘common’ group (*i.e.* shared annotated peaks identified in unstimulated and TNF-stimulated; n = 12,446 genes, from a total of 22,016 annotated peaks) revealed 216 genes overlapping both sequencing sets. Comparison with peaks unique only the TNF stimulated group (n = 688 genes, from 709 annotated peaks) highlighted 25 genes common to both sequencing sets; see **Supplementary Information—Figure S2**.

### NF*κ*B Target Genes Induced by TNF

Gene ontology enrichment analysis of the early phase genes showed the NF*κ*B pathway among the most important signalling pathways identified (**Supplementary Information—Table S3**). Therefore, all significant differentially expressed genes (n = 1,141) were further analysed against reference NF*κ*B target gene data (**Supplementary Information—Figure S3**). At 0.5 h, all six genes upregulated in response to 40 ng/ml TNF were identified as NF*κ*B target genes, including *Tnf*, *Nfkbia*, *Tnfaip3*, *Nfkbiz*, *Irf1*, and *Ccl20*. It is interesting to point out that these NF*κ*B target genes, apart from *Ccl20*, formed the early phase response to TNF, as identified in Cluster 7 (**Figure 3**). The proportion of NF*κ*B target genes decreased over time, representing 100 (6/6), 47.4 (9/19), 13.4 (68/509), and 10.6% (97/915) of total differentially expressed genes at 0.5, 1, 2, and 24 h post-TNF stimulation, respectively (see **Supplementary Information—Figure S3**). The majority of the NF*κ*B target genes were upregulated upon TNF treatment, with a smaller fraction being downregulated in the two later time points, representing 23.5% (16/68) at 2 h, and 26.8% (26/97) at 24 h, respectively. Among those genes identified was *Il10rb*, encoding the beta subunit of the IL-10 receptor (44). Transcription factor enrichment analysis revealed NF*κ*B family subunits as the major regulators of the immediate–early response genes induced by TNF at 0.5 and 1 h. Analysis also revealed enrichment in members of the STAT family of transcription factors, such as STAT1*α*/3/4/5*α*/6; (see **Supplementary Information—Table S4**).

To validate TNF-induced NF*κ*B target genes of significance identified from the RNA sequencing, fresh C57BL/6J enteroids were stimulated with 40 ng/ml TNF for 1 h, with total RNA extracted and qPCR analysis performed on reverse transcribed cDNA. We assessed expression levels for the early phase genes represented within Cluster 7; *Tnf*, *Tnfaip3, Nfkbia*, *Irf1*, and *Nfkbiz* (see **Supplementary Information—Figure S4**). All five genes showed statistically significant upregulation upon TNF treatment; the highest being *Tnf* and the lowest being *Nfkbiz* (*p* < 0.05, Kruskal–Wallis test; N = 3). In parallel studies, we treated enteroids with 100 ng/ml flagellin for 1 h. Whilst flagellin was a less potent activator of enteroids than TNF, similar expression profiles for all five genes studied were seen (**Supplementary Information—Figure S4**).

### NF*κ*B Target Genes Are Differentially Expressed in TNF- and Flagellin-Stimulated Enteroids Deficient in Interleukin 10

**Figure 4:**
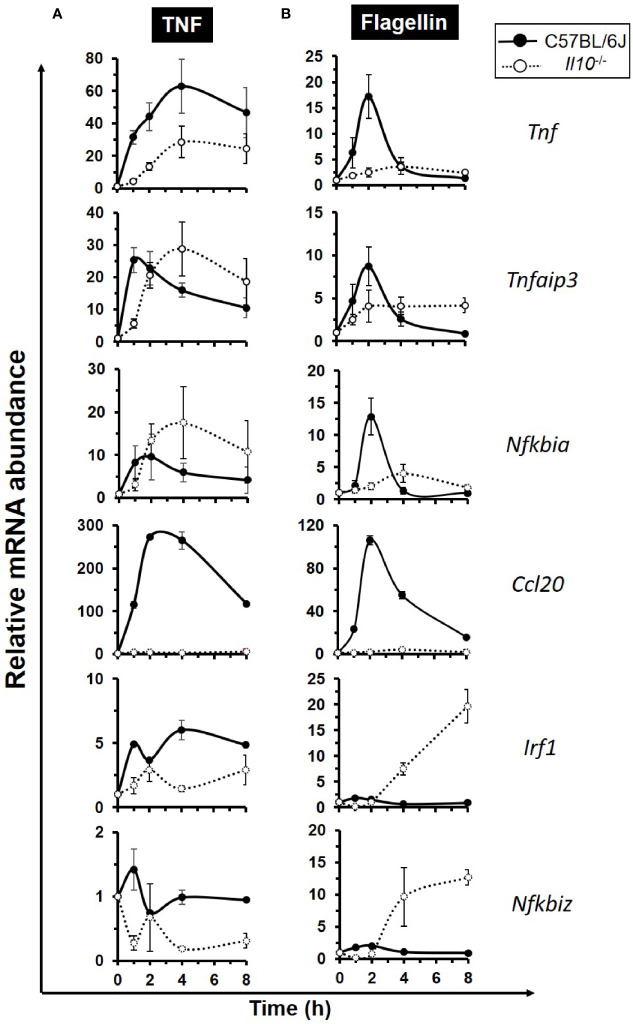
Defective expression of the early response NF*κ*B target genes in interleukin 10 deficient enteroids. Enteroid cultures from C57BL/6J and *Il10^−/−^* mice were left unstimulated or stimulated with either **(A)** 40 ng/ml TNF, or **(B)** 100 ng/ml flagellin, for up to 8 h. qPCR analysis of early response NF*κ*B target genes *Tnf*, *Tnfaip3*, *Nfkbia*, *Ccl20*, *Irf1*, and *Nfkbiz* was performed. Data is presented as mean ± SEM; N = 3–4 mice.

To examine whether IL-10 deficiency impacted on intestinal epithelium-specific expression of NF*κ*B target genes, enteroids from both C57BL/6J and *Il10^−^* ^/^ *^−^* mice were either left unstimulated or they were stimulated with 40 ng/ml TNF, over 8 h. At rest, *Tnf* mRNA levels were five-fold higher in *Il10^−^* ^/^ *^−^* enteroids compared to controls (*p* < 0.05, Kruskal–Wallis test, N = 3–4 mice). Absolute levels of all other genes tested were low, with *Nfkbia, Nfkbiz*, and *Irf* in *Il10^−^* ^/^ *^−^* enteroids significantly reduced (all *p* < 0.05); see **Supplementary Information—Figure S5**. Notably, TNF induced expression of its own gene in both genotypes, with the response markedly reduced in IL-10 deficient enteroids (45.3% reduction at peak time 4 h; *p* < 0.05, N = 3–4 mice), **Figure 4A**. Similarly, reduced response to TNF was also observed for *Irf1*, *Nfkbiz*, and *Ccl20*, the latter of which was almost undetectable in *Il10^−^* ^/^ *^−^* enteroids. Attenuated early phase response to TNF (1 h) in *Il10^−^* ^/^ *^−^* enteroids was also seen for both *Nfkbia* and *Tnfaip3*, although their mRNA levels were much higher at later time points (4–8 h) than those of the wild-type (**Figure 4A**). Parallel cultures of 100 ng/ml flagellin-treated *Il10^−^* ^/^ *^−^* enteroids (N = 3–4 mice) showed similar defect in *Tnf* and *Ccl20* expression (**Figure 4B**), albeit expression levels were considerably lower compared to those seen following TNF stimulation. Flagellin-induced *Nfkbia* and *Tnfaip3* early phase expression was also suppressed in the absence of IL-10, with delayed elevation of expression at later time points (**Figure 4B**). Similarly, *Il10^−/−^* enteroids show attenuated expression of early phase genes *Irf1* and *Nfkbiz* upon flagellin stimulation but markedly higher expression ≥2 h post-treatment. In addition, *Tnip1* and *Tnip2*, genes encoding ABIN proteins reported to interact with A20 and regulate the NF*κ*B pathway (59, 60) were also examined (**Supplementary Information—Figure S6**). At rest *Tnip1* levels were significantly lower in IL-10 deficient enteroids (*p* < 0.05). Only *Tnip1* was significantly induced by TNF, and to a much lesser extent by flagellin, compared to unstimulated controls. However, both *Tnip1* and *Tnip2* levels were elevated by both stimuli in *Il10^−^* ^/^ *^−^* enteroids compared to C57BL/6J controls, particularly at later time points.

### IL-10 Expression in TNF-Induced C57BL/6J Enteroids

**Figure 5:**
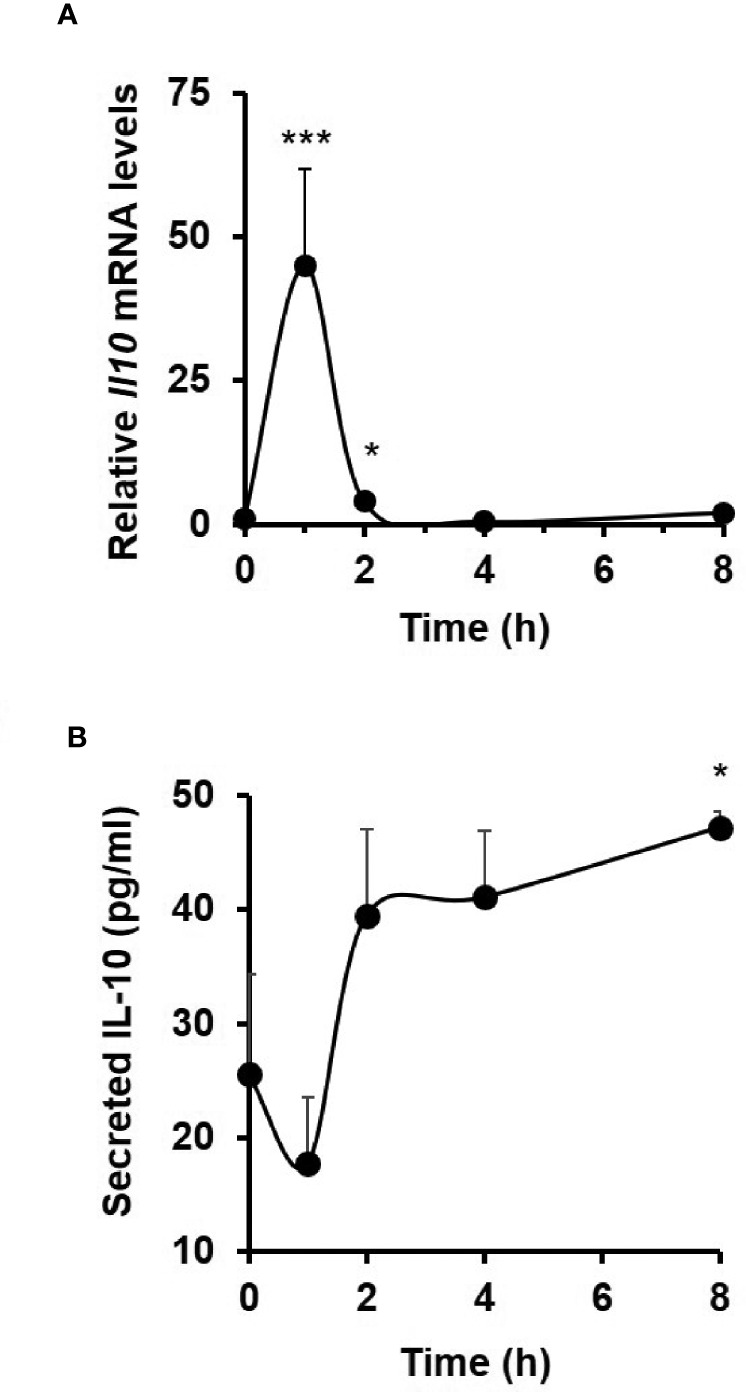
TNF induces interleukin 10 expression and release from C57BL/6J enteroids. Analysis of **(A)** *Il10* mRNA (measured by qPCR) and **(B)** IL10 protein (measured by ELISA) in resting and 40 ng/ml TNF-treated enteroid cultures from C57BL/6J mice. Significant differences compared to untreated controls, *p < 0.05 and ***p < 0.001; Kruskal–Wallis test. Data is presented as mean ± SEM; N = 6 mice.

In response to 40 ng/ml TNF treatment, *Il10* mRNA levels in C57BL/6J enteroid cultures were seen to rapidly peak at 1 h (44.9 ± 16.9-fold increase; *p* < 0.001, Kruskal–Wallis test; N = 6 mice), with a return to resting levels by 4 h (**Figure 5A**). TNF-induced secretion of IL-10 protein to culture medium was observed at 2 h, with levels at 8 h reaching 47.2 ± 1.4 pg/ml (mean ± SEM) compared to untreated controls; *p* < 0.05; N = 6 mice (**Figure 5B**).

### The IL-10/IL-10R Axis Impacts on Endogenous NF*κ*B Activation and NF*κ*B Target Gene Transcription

**Figure 6:**
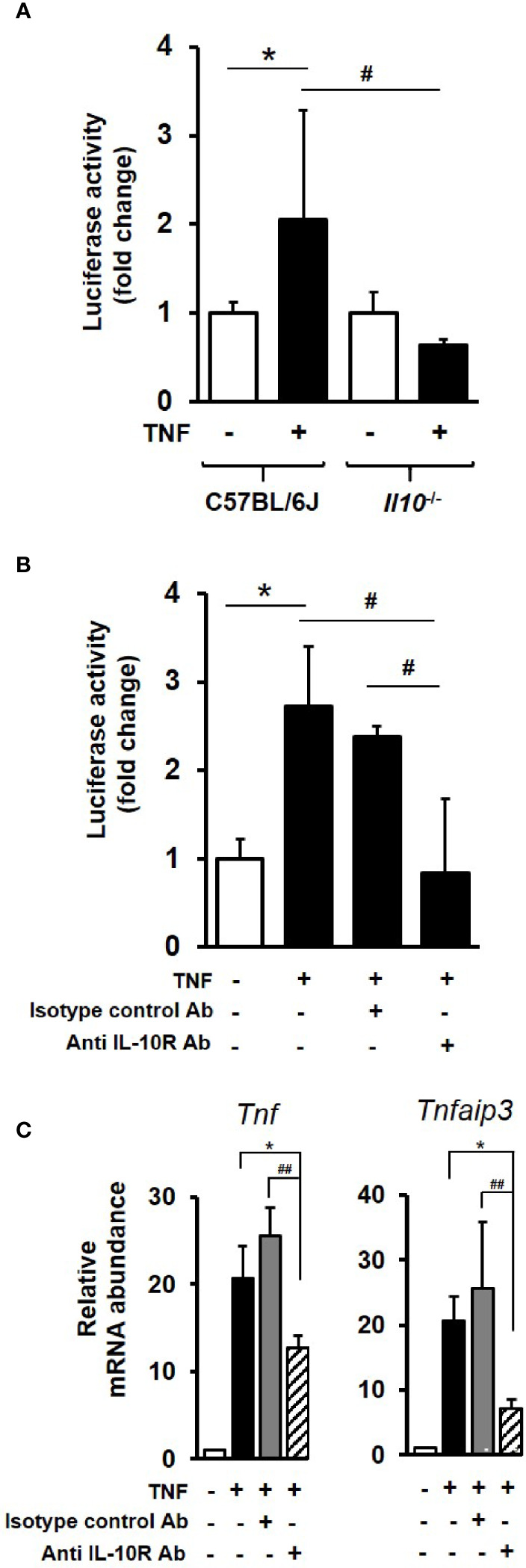
IL-10 deficiency or IL-10R signaling blockade impacts on endogenous NFκB activation and downstream gene transcription in enteroids. **(A)** C57BL/6J and *Il10^−/−^* enteroids were transduced with lentivirus that expresses luciferase under the control of canonical NFκB binding sequence motif. Post transduction (72 h), enteroids were either left unstimulated or stimulated with 100 ng/ml TNF for 3 h. Luminescence was detected using single tube luminometer. **(B)** Immunoblockade of hTNF.LucBAC enteroids using 1 μg/ml rat anti-mouse IL-10 receptor (IL-10R) antibody (Ab) or rat IgG1, *κ* isotype control, followed by 2 h stimulation with 100 ng/ml TNF. Significant differences to unstimulated control, *p < 0.05; or stimulated transgenic or immunoblockade, ^#^ *p* < 0.05, Kruskal–Wallis test; N = 3–4 mice). **(C)** Immunoblockade using anti-IL-10R antibody also significantly suppressed TNF-induced expression at 1 h of two selected early response NF*κ*B target genes *Tnf* and *Tnfaip3* in C57BL/6 enteroids. Significant differences, *p < 0.05 TNF plus IL-10R antibody compared to enteroids stimulated with TNF alone, and ^##^ *p* < 0.01 compared to TNF plus isotype control antibody; Kruskal–Wallis test, N = 3 mice. All data is presented as mean ± SEM.

To examine the impact of IL-10 deficiency on NF*κ*B activity, enteroids were transduced with lentivirus that expresses luciferase under the promoter of the p50/65 heterodimer. Transduced C57BL/6J enteroids stimulated for 3 h with 100 ng/ml TNF, showed a 2.1-fold increase in NF*κ*B-regulated luciferase activity compared to unstimulated controls (*p* < 0.05; Kruskal–Wallis test; N = 3 mice), whereas no response to treatment was observed in IL-10 deficient enteroids (**Figure 6A**). In an additional experiment performed using hTNF.LucBAC enteroids expressing luciferase under the control of the human TNF promoter, 100 ng/ml TNF for 2 h induced a 2.7-fold increase in signal over 2 h compared to unstimulated controls (*p* < 0.05; Kruskal–Wallis test; N = 3–4 mice). Pre-treatment of C57BL/6J enteroids overnight with 1 μg/ml anti-mouse IL-10R antibody significantly blocked TNF-induced luciferase activity (*p* < 0.05). No such suppression observed using the isotype control (**Figure 6B**).

At the level of gene transcription, immunoblockade of IL-10R signalling in enteroids was observed to markedly suppress TNF-induced expression of early response NF*κ*B target genes *Tnf*, (39.0% reduction in transcription) and *Tnfaip3* (65.1% reduction); both *p* < 0.05 compared to enteroids stimulated with TNF alone, and *p* < 0.01 compared to TNF plus isotype control antibody; N = 3 mice. No significant suppression of TNF-induced increase in *Tnf* or *Tnfaip3* transcription was observed using the isotype control antibody*;* see **Figure 6C**.

**Figure 7:**
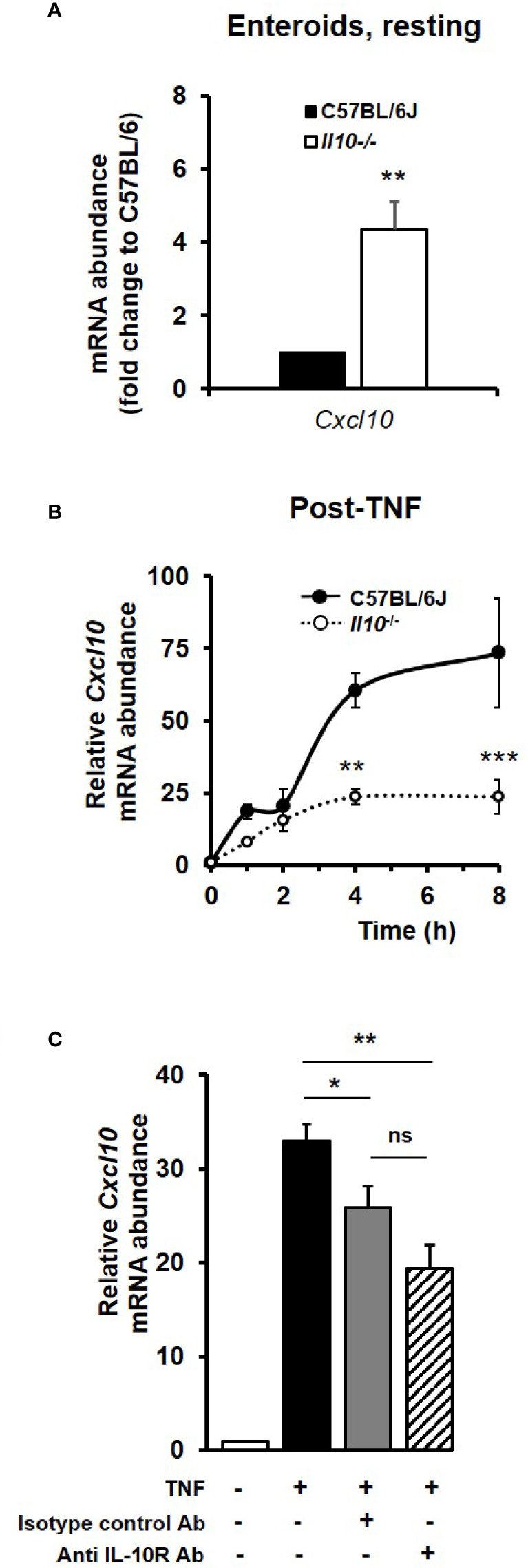
The TNF-induced late response NF*κ*B target gene *Cxcl10* is dependent on IL-10/IL-10R signaling. *Cxcl10* mRNA levels in enteroid cultures from C57BL/6J and *Il10^−/−^* mice, **(A)** at rest, and **(B)** following treatment for 8 h with 40 ng/ml TNF. Significant differences of Il10 null enteroids compared to C57BL/6J controls, **p < 0.01 and ***p < 0.001 (N=3-4 mice, Kruskal–Wallis test). **(C)** Assessment of *Cxcl10* mRNA levels (as measured by qPCR) following overnight immunoblockade with rat anti-mouse IL-10 receptor (IL-10R) antibody (Ab) or using rat IgG1, *κ* isotype control, stimulated with 40 ng/ml TNF for 8 h. Significant differences compared to enteroids stimulated with TNF alone as per Kruskal–Wallis test; *p < 0.05 and **p < 0.01. ns, no significant difference between TNF plus anti-IL-10R and TNF plus isotype control antibody treatments (*p* = 0.18). All data presented as mean ± SEM; N = 3–4 mice.

Similarly, C–X–C motif chemokine 10 (*Cxcl10*), a key late response NF*κ*B target gene identified within the RNA sequencing data set as being significantly induced by TNF, was also shown to be regulated by the IL-10/IL-10R axis in enteroid cultures. At rest, *Cxcl10* mRNA levels were observed to be 4.35 (± 0.77) fold higher in *Il10^−^* ^/^ *^−^* enteroids compared to controls (*p* < 0.01, Kruskal–Wallis test, N = 4 mice); see **Figure 7A**. TNF, at 40 ng/ml, increased the expression of *Cxcl10* measured through to 8 h, with the response seen to be significantly reduced in enteroids deficient in IL-10 (67.8% reduction at peak time 8 h; *p* < 0.001, N = 3–4 mice); see **Figure 7B**. Immunoblockade of IL-10R in C57BL/6J enteroids was also seen to markedly suppress TNF-induced increase in *Cxcl10* mRNA levels by 41.2%; *p* < 0.01, N = 3 mice (**Figure 7C**). However, whilst this was significant compared to TNF treatment alone, it was not significant when compared to TNF plus isotype control antibody (*p* = 0.18), which was also seen to significantly reduce *Cxcl10* mRNA levels by 21.7% compared to TNF alone (*p* < 0.05) (**Figure 7C**).

## Discussion

*In vitro* cultures of intestinal crypt stem cell-derived epithelial organoids are now widely used for studies on the physiological responses of the intestinal epithelium (61, 62). These cultures can be maintained long-term due to the presence of stem cells that have the ability, under specific stimuli, to give rise to epithelial cell subpopulations with specific functions (63, 64). Moreover, mammalian intestinal epithelial organoids have been shown to have epigenetic signatures with striking similarities to the ones of the original tissue (65), offering advantages to research transcriptional regulation within the intestinal epithelium. One such approach is ATAC sequencing, a genome-wide open chromatin analysis that reflects active transcription and epigenetic modifications, with recent reports of its use with human intestinal organoids (66). Here, we have established an ATAC sequencing protocol for use with murine enteroids in order to study the transcriptional effects of TNF. Our analysis revealed that a significant number of genes following stimulation with TNF were seen to have open chromatin, particularly in their promotor regions, including *Tnf* and *Btg2* (B cell translocation gene 2), both known to be important in the intestinal epithelium (67, 68). We also identified other key genes that had open chromatin at resting levels in enteroids, with additional peaks seen upon TNF stimulation, including genes associated with the NFκB signalling pathway and downstream NF*κ*B target genes (60, 69). Pathway enrichment analysis of the promoter region-related peaks showed that the integrin signalling pathway was also highly represented and known to regulate apoptotic processes in the intestinal epithelium (70).

For further in-depth analysis of the transcriptional effects of TNF, we performed quantitative RNA sequencing on enteroid cultures stimulated for up to 24 h. Analysis here identified over 1,000 differentially expressed genes throughout the time course, with the majority being upregulated compared to the unstimulated controls, especially within the first two sampling time points at 0.5 and 1 h. Cluster analysis assigned genes to seven groups based on their expression profile, from early phase genes peaking at 1 h (Cluster 7), to late response genes that peak in expression at 2 h, reaching a plateau through to 24 h (Cluster 4), or continuing to increase in their expression (Clusters 5 and 6). Gene ontology enrichment analysis performed on all differentially regulated genes indicated that the most prominent pathway enriched was the NF*κ*B transcription factor pathway. A strikingly high proportion of NF*κ*B target genes was identified in the early time points, 100 and 47% at 0.5 and 1 h, respectively. This was further supported by transcription factor enrichment analysis, which also revealed NF*κ*B family subunits as the major regulators of the immediate–early response genes induced by TNF. Our data support previous observations, that TNF impacts on intestinal homeostasis by activating NF*κ*B signalling and that this can promote cell survival or cell death, dependent on duration of the stimulation and/or cellular condition (71, 72). Amongst those early phase NF*κ*B targets identified by RNA sequencing, were genes we had already shown as having open chromatin loci using ATAC sequencing. One example was *Tnf*, a gene whose promoter region contains a known NF*κ*B binding motif which upon transcription factor binding, results in positive autoregulation of its own transcription (73). We showed other TNF-induced early response genes (e.g. *Tnfaip3*, *Nfkbia* and *Irf1*) to have open chromatin under resting conditions. Previous RNA sequencing studies in murine enteroids also support these observations for *Tnfaip3* (74, 75)*, Nfkbia* (74, 75), and *Irf1* (74) expression under resting conditions. For *Nfkbiz* and *Ccl20* however, we found these genes to have open chromatin evident at rest but with little detectable expression, until that is, following exposure to TNF. Other studies also support negligible levels of expression of these two genes in enteroids under basal conditions (74, 75). This suggests that these might be considered as ‘poised’ genes (*i.e.* genes whose promotors are preloaded with poised polymerase II to prepare for rapid activation (76, 77). Further studies to examine these loci at the chromatin level would be necessary to support this hypothesis. In addition, we noted that both *Tnfaip3* and *Tnip1*, encoding for A20 and ABIN1 respectively, appeared in both high-throughput sequencing approaches. These two proteins have been shown to be upregulated by TNF and synergistically prevent intestinal inflammation by restricting intestinal epithelial death and preserving tissue integrity (78). One of the downregulated genes identified was *Il10rb*, encoding the signalling subunit of the IL-10 receptor (3, 44).

It is well established that in immune cells, IL-10 can inhibit NF*κ*B activation and its subsequent proinflammatory action (3, 24, 25), and this was further supported recently by RNA sequencing analysis of LPS-induced *Il10* deficient macrophages (79). Little is known though, about the function of the intestinal epithelium-produced IL-10, which was the focus of our study here. Previous *in vivo* studies have suggested a protective role for IL-10 in preventing intestinal inflammation, with *Il10rb^−^* ^/^ *^−^* mice developing spontaneous colitis (3). In addition, a comparative study of IBD and a piroxicam-accelerated colitis *Il10^−^* ^/^ *^−^* model shows a high degree of similarity in their colon transcriptional signatures (80). In these *in vivo* models though, it is almost impossible to separate autocrine *versus* paracrine effects and understand how each individual cell type functions within the immune and epithelial cell compartments.

However, using our *in vitro* enteroid culture system, we were able to perform experiments to monitor the dynamic transcriptional response of TNF in both wild-type and *Il10* null enteroids. We were also able to examine the action of bacterial flagellin, known to activate the NF*κ*B pathway downstream of TLR5 (81) and play an important role in the development and progression of intestinal inflammation (82). Quantitative qPCR validated our RNA sequencing data, and clearly showed an intrinsic, intestinal epithelium-specific effect of IL-10 deficiency on gene transcription. This was also confirmed following immunoblockade of IL-10R in wild-type enteroids. Flagellin also significantly induced expression of *Tnf*, *Nfkbia*, *Tnfaip3*, and *Ccl20* in agreement with previous RNA sequencing data from flagellin-induced of murine organoids (74). Absence of IL-10 attenuated both TNF- and flagellin-induced expression of the early phase genes within the first hour, including *Tnf* which was markedly reduced and *Ccl20*, whose gene transcription was effectively abolished. The dynamics of *Tnfaip3* and *Nfkbia* transcription were similar in both genotypes, but elevated in *Il10* null enteroids. IL-10 appeared to be a negative regulator of *Tnfaip3* and *Nfkbia*, both encoding known inhibitors of NF*κ*B (54, 55), as their expression levels were observed in our study to be higher or sustained for a longer period in IL-10 deficient enteroids compared to the wild-type cultures. With flagellin, we observed enhanced transcription of *Irf1* and *Nfkbiz* in IL-10 null enteroids. This is perhaps not unexpected since the two receptors do not share identical downstream signalling pathways, with TNFR1 activating receptor interacting protein (RIP) and the TNF receptor-associated factor 2 (TRAF2) pathway (71), and TLR5 signalling utilising the MyD88 adaptor (83). As has been reported previously, there are quantitative and gene-specific differences in responses towards the two stimuli (84). For flagellin, our data support IL-10 as exerting part of its anti-inflammatory function by negatively regulating the levels of Irf1 in the intestinal epithelium. Irf1 regulates the expression of C–X–C motif chemokine 10 (Cxcl10), that acts as a chemoattractant for immune cells and promotes inflammation (69). Within our RNA sequencing dataset, we observed *Cxcl10* to be a TNF-induced late response NF*κ*B target gene, and its transcription was found to significantly suppressed in IL-10 deficient enteroids. Cxcl10 expression is reported as being localised mainly to epithelial cells in the intestinal mucosa, with increased levels seen in IBD tissues (85), where mucosal levels of pro-inflammatory TNF are also notably high (27). Although our *in vitro* enteroid data contradict the anti-inflammatory role of IL-10 in relation to *Cxcl10* expression following TNF stimulation, further *in vitro* and *in vivo* experiments are necessary in order to understand the regulation of this late response gene. This is because there are clear limitations of the enteroid model which do not represent the complex interplay for immune and stromal cells that would be found *in vivo*. Also, it is known that *Cxcl10* transcription depends on protein synthesis (86, 87) and on additional transcription factors, such as activator protein 1 (AP-1) (87) and key transcriptional repressors 4E-BP1/2 (88).

To further establish a role for epithelium-derived IL-10, we examined for its expression in wild-type enteroids. We showed early TNF-induced transcription of *Il10*, which was followed by low levels of IL-10 protein detected in the enteroid culture medium, at picogram levels similar to those previously reported in small intestinal tissue from C57BL/10 mice (89). We then monitored the activation of the NF*κ*B pathway upon TNF stimulation in the absence of IL-10 or immunoblockade of the IL-10R. Utilising transduced and transgenic luciferase reporter enteroids, our data clearly showed that IL-10 is a positive regulator of epithelium-specific endogenous NF*κ*B activation. A key study supporting our data is that conducted previously by Nenci and colleagues, where they demonstrated that intestinal-specific ablation of the upstream kinases of the canonical NF*κ*B pathway resulted in development of spontaneous, severe intestinal inflammation in mice (22). These data clearly suggest the NF*κ*B signalling pathway in the gut epithelium as a critical regulator of epithelial integrity and intestinal immune homeostasis (90). Anti-inflammatory function of epithelial-derived IL10 through autocrine action *via* the non-classical MHC molecule CD1d has also been proposed in another recent study, where IL-10 is shown to attenuate IFN*γ*-induced intestinal epithelium damage (91). The importance of IL-10 signalling on the maintenance and restitution of intestinal epithelial barrier is further highlighted in *in vivo* studies using IL-10 receptor deficient mice (92). Moreover, IL-10 receptor point mutations have been linked to inflammatory conditions, such as IBD (93, 94).

In summary, our study utilised ATAC and RNA sequencing and *in vitro* enteroid culture approaches to study the (patho)physiological response of the intestinal epithelium to TNF. We have shown that upon stimulation, the NF*κ*B pathway is the major transcriptional regulator of the intestinal epithelium. Moreover, epithelium-derived IL-10 appears to play a crucial autocrine role in positively regulating NF*κ*B and therefore supporting its function as a major regulator of intestinal homeostasis. Dysregulation of such a potent cytokine could potentially lead to a low level, chronic inflammation underlying intestinal epithelial disease. We have recently reported a recombinant stable IL-10 dimer that attenuates bacterial lipopolysaccharide-induced dermal inflammation (95), thus highlighting the potential for future tissue-targeted IL-10 therapeutic intervention.

## Data Availability Statement

The datasets presented in this study can be found in online repositories. The names of the repository/repositories and accession number(s) can be found below: https://www.ebi.ac.uk/arrayexpress/, E-MTAB-10217 and https://www.ebi.ac.uk/ena, PRJE43395.

## Ethics Statement

The animal study was reviewed and approved by Home Office of the United Kingdom-Home Office Licence (PPL: 70/8457).

## Author Contributions

BC, PR, SP, VMDS and WM: conception, design and obtainment of funding. CW, LP, PR, SP, SS, RS-T and WT: acquisition of data. BC, FB, LL, LP, MA, RS-T, SP, SS, VMDS, WM and WT: analysis and interpretation of data. BC, FB, LL, MA and VMDS: bioinformatics and statistical analyses. BC and SP: drafted the manuscript. All authors contributed to the article and approved the submitted version.

## Funding

Studies were initiated under the SysmedIBD project (www.sysmedibd.eu/) with funding support from the European Community Seventh Framework Programme (FP7—Health; 2007-2013) under grant agreement ID number 305564. SP was supported by a Career Development Award scheme supported by the Technology Directorate, University of Liverpool and a Wellcome Trust Institutional Strategic Support Fund (ISSF) award to the Faculty of Health and Life Sciences, University of Liverpool (204822/Z/16/Z). LP and BC were supported by The Wellcome Trust through the 4-year PhD programme in Molecular & Cellular Physiology, at the University of Liverpool (102172/B/13/Z). Interleukin 10 knockout (*Il10^−/−^*) mice were available through an award to BC from the Medical Research Council (MR/P023606/1). The funders provided no input to the study design nor in the collection, analyses and interpretation of data.

## Conflict of Interest

VMDS is a director and shareholder of LifeGlimmer GmbH. FB has received salary from LifeGlimmer GmbH.

The remaining authors declare that the research was conducted in the absence of any commercial or financial relationships that could be construed as a potential conflict of interest.

## Supplementary Material

Supplementary Table 1 Gene ontology enrichment of the TNF-induced sample.

Supplementary Table 2 RNA sequencing cluster analysis of TNF-induced enteroids - gene names and relative fold changes to unstimulated controls.

Supplementary Table 3 Gene ontology enrichment of the early phase targets identified by RNA sequencing.

Supplementary Table 4 Transcription factor enrichment analysis of the immediate-early response genes induced by TNF.

Supplementary Figure 1 Tagmentation fragments and qPCR analysis.

Supplementary Figure 2 Proportion of TNF-induced genes identified as being common to both ATAC and RNA sequencing data sets.

Supplementary Figure 3 Proportion of genes identified as NFkB target genes from TNF-stimulated enteroid RNA sequencing.

Supplementary Figure 4 qPCR analysis of early response NFkB regulated genes in C57BL/6J enteroids.

Supplementary Figure 5 Resting levels of NFkB target genes in enteroids deficient in interleukin 10.

Supplementary Figure 6 NFkB target genes encoding ABIN1 and ABIN2 are overexpressed in interleukin 10 deficient enteroids.
