# Supplementary material for: Impact of Interleukin 10 Deficiency on Intestinal Epithelium Responses to Inflammatory Signals
Source: Front Immunol. 2021 Jun 16;12:690817. doi: 10.3389/fimmu.2021.690817 (PMC8244292; doi:10.3389/fimmu.2021.690817)
Supplement: Supplementary Table 1 — Gene ontology enrichment of the TNF-induced sample. [file Table_1.docx]

**SUPPLEMENTARY INFORMATION**

**Table S1. Gene ontology enrichment of the TNF-induced sample.** Informatic analysis of the ATAC sequencing data identified peaks in various genomic locations. Peaks that appeared in the promoter region of genes in the TNF-induced sample were used for gene ontology (GO) enrichment analysis, as described under Materials and Methods.

| **GO molecular function complete** | ***Mus. musculus***  **- REFLIST (21988)** | **Promoter (602)** | **Raw**  ***p*-value** | **FDR**  ***p*<0.05** |
| --- | --- | --- | --- | --- |
| protein binding (GO:0005515) | 9310 | 335 | 1.03E-10 | 4.80E-07 |
| olfactory receptor activity (GO:0004984) | 1134 | 3 | 2.83E-10 | 6.59E-07 |
| binding (GO:0005488) | 13720 | 444 | 6.98E-09 | 1.08E-05 |
| anion binding (GO:0043168) | 2734 | 124 | 3.49E-08 | 4.05E-05 |
| GTPase binding (GO:0051020) | 522 | 39 | 5.77E-08 | 5.36E-05 |
| small GTPase binding (GO:0031267) | 424 | 34 | 8.87E-08 | 6.87E-05 |
| enzyme binding (GO:0019899) | 2367 | 109 | 1.36E-07 | 9.05E-05 |
| protein kinase binding (GO:0019901) | 746 | 47 | 3.98E-07 | 2.05E-04 |
| Ras GTPase binding (GO:0017016) | 409 | 32 | 3.54E-07 | 2.06E-04 |
| kinase binding (GO:0019900) | 833 | 50 | 5.75E-07 | 2.67E-04 |
| Rho GTPase binding (GO:0017048) | 168 | 19 | 6.72E-07 | 2.84E-04 |
| ion binding (GO:0043167) | 5508 | 205 | 1.16E-06 | 4.47E-04 |
| guanyl-nucleotide exchange factor activity (GO:0005085) | 201 | 20 | 2.14E-06 | 7.65E-04 |
| calcium ion binding (GO:0005509) | 616 | 39 | 3.15E-06 | 1.05E-03 |
| transporter activity (GO:0005215) | 1110 | 58 | 5.67E-06 | 1.76E-03 |
| 14-3-3 protein binding (GO:0071889) | 31 | 8 | 7.20E-06 | 2.09E-03 |
| carbohydrate derivative binding (GO:0097367) | 2131 | 94 | 7.88E-06 | 2.15E-03 |
| protein-containing complex binding (GO:0044877) | 1471 | 70 | 1.19E-05 | 3.07E-03 |
| adenyl ribonucleotide binding (GO:0032559) | 1456 | 69 | 1.58E-05 | 3.87E-03 |
| ribonucleotide binding (GO:0032553) | 1805 | 81 | 1.83E-05 | 4.24E-03 |
| adenyl nucleotide binding (GO:0030554) | 1468 | 69 | 2.42E-05 | 4.49E-03 |
| purine ribonucleotide binding (GO:0032555) | 1788 | 80 | 2.35E-05 | 4.54E-03 |
| ATP binding (GO:0005524) | 1390 | 66 | 2.26E-05 | 4.56E-03 |
| Ras guanyl-nucleotide exchange factor activity (GO:0005088) | 125 | 14 | 2.16E-05 | 4.56E-03 |
| GTPase regulator activity (GO:0030695) | 258 | 21 | 2.11E-05 | 4.67E-03 |
| enzyme activator activity (GO:0008047) | 462 | 30 | 2.71E-05 | 4.85E-03 |
| purine ribonucleoside triphosphate binding (GO:0035639) | 1712 | 77 | 3.15E-05 | 5.42E-03 |
| purine nucleotide binding (GO:0017076) | 1801 | 80 | 3.44E-05 | 5.51E-03 |
| transmembrane transporter activity (GO:0022857) | 1012 | 52 | 3.35E-05 | 5.55E-03 |
| nucleoside-triphosphatase regulator activity (GO:0060589) | 299 | 22 | 5.62E-05 | 8.71E-03 |
| GTPase activator activity (GO:0005096) | 238 | 19 | 6.56E-05 | 9.83E-03 |
| Rho guanyl-nucleotide exchange factor activity (GO:0005089) | 73 | 10 | 7.10E-05 | 1.03E-02 |
| small molecule binding (GO:0036094) | 2433 | 99 | 1.03E-04 | 1.46E-02 |
| nucleotide binding (GO:0000166) | 2032 | 85 | 1.22E-04 | 1.62E-02 |
| nucleoside phosphate binding (GO:1901265) | 2032 | 85 | 1.22E-04 | 1.67E-02 |
| ion transmembrane transporter activity (GO:0015075) | 840 | 43 | 1.59E-04 | 2.05E-02 |
| ATPase-coupled transmembrane transporter activity (GO:0042626) | 101 | 11 | 2.03E-04 | 2.54E-02 |
| transmembrane signaling receptor activity (GO:0004888) | 2140 | 33 | 2.53E-04 | 3.01E-02 |
| gated channel activity (GO:0022836) | 324 | 22 | 2.48E-04 | 3.03E-02 |
| metal ion transmembrane transporter activity (GO:0046873) | 424 | 26 | 2.70E-04 | 3.13E-02 |
| primary active transmembrane transporter activity (GO:0015399) | 107 | 11 | 3.20E-04 | 3.63E-02 |
| actin filament binding (GO:0051015) | 210 | 16 | 3.93E-04 | 4.25E-02 |
| inorganic molecular entity transmembrane transporter activity (GO:0015318) | 795 | 40 | 3.91E-04 | 4.33E-02 |
| ion channel activity (GO:0005216) | 420 | 25 | 4.84E-04 | 4.89E-02 |
| molecular_function (GO:0003674) | 20176 | 575 | 4.81E-04 | 4.97E-02 |
| Unclassified (UNCLASSIFIED) | 1812 | 27 | 4.81E-04 | 5.08E-02 |

*FDR, false discovery rate*
