# Supplementary material for: Impact of Interleukin 10 Deficiency on Intestinal Epithelium Responses to Inflammatory Signals
Source: Front Immunol. 2021 Jun 16;12:690817. doi: 10.3389/fimmu.2021.690817 (PMC8244292; doi:10.3389/fimmu.2021.690817)
Supplement: Supplementary Table 3 — Gene ontology enrichment of the early phase targets identified by RNA sequencing. [file Table_3.docx]

**SUPPLEMENTARY INFORMATION**

**Table S3. Gene ontology enrichment of the early phase targets identified by RNA sequencing.** Statistically significant genes identified within the 1h of stimulation, were subsequently filtered for ± 2-fold changes. The resulting gene list was submitted for gene ontology enrichment analysis on Panther system as described within the Materials and Methods.

| **PATHWAYS** | ***Mus. musculus***  **REFLIST** | **RNAseq**  **genes** | **Fold enrichment** | **Raw**  ***p*-value** | **FDR**  **(*p*<0.05)** |
| --- | --- | --- | --- | --- | --- |
| Death Recepto+B13:G24r Signaling (R-MMU-73887) | 128 | 4 | 40.42 | 2.77E-06 | 2.28E-03 |
| DDX58/IFIH1-mediated induction of interferon-alpha/beta (R-MMU-168928) | 32 | 3 | > 100 | 2.47E-06 | 4.07E-03 |
| ZBP1(DAI) mediated induction of type I IFNs (R-MMU-1606322) | 13 | 2 | > 100 | 5.86E-05 | 1.61E-02 |
| RIP-mediated NFkB activation via ZBP1 (R-MMU-1810476) | 12 | 2 | > 100 | 5.08E-05 | 1.68E-02 |
| TRAF6 mediated NF-kB activation (R-MMU-933542) | 15 | 2 | > 100 | 7.59E-05 | 1.79E-02 |
| TNFR1-induced proapoptotic signaling (R-MMU-5357786) | 12 | 2 | > 100 | 5.08E-05 | 2.10E-02 |
| SUMOylation of immune response proteins (R-MMU-4755510) | 11 | 2 | > 100 | 4.36E-05 | 2.40E-02 |
| Regulation of TNFR1 signaling (R-MMU-5357905) | 33 | 2 | 78.39 | 3.29E-04 | 4.52E-02 |
| Ovarian tumor domain proteases (R-MMU-5689896) | 32 | 2 | 80.84 | 3.11E-04 | 4.66E-02 |
| TAK1 activates NFkB by phosphorylation and activation of IKKs complex (R-MMU-445989) | 30 | 2 | 86.23 | 2.75E-04 | 5.04E-02 |
| Cytosolic sensors of pathogen-associated DNA (R-MMU-1834949) | 32 | 2 | 80.84 | 3.11E-04 | 5.12E-02 |
| TNFR1-induced NFkappaB signaling pathway (R-MMU-5357956) | 29 | 2 | 89.2 | 2.58E-04 | 5.31E-02 |

*FDR, false discovery rate*
