# Supplementary material for: Impact of Interleukin 10 Deficiency on Intestinal Epithelium Responses to Inflammatory Signals
Source: Front Immunol. 2021 Jun 16;12:690817. doi: 10.3389/fimmu.2021.690817 (PMC8244292; doi:10.3389/fimmu.2021.690817)
Supplement: Supplementary Table 4 — Transcription factor enrichment analysis of the immediate-early response genes induced by TNF. [file Table_4.docx]

**SUPPLEMENTARY INFORMATION**

**Table S4. Transcription factor enrichment analysis of the immediate-early response genes induced by TNF.** Statistically significant genes identified within 0.5h and 1h of stimulation, were subsequently filtered for ± 2-fold changes. The resulting gene list was analysed using the PASTAA tool at <http://trap.molgen.mpg.de> to enable prediction of affinities of common transcription factors to the promoters of those genes, as described under Materials and Methods. A cut of *P*<0.05 was used, which generated a final list of 50 ranked predictions as shown.

| **Rank** | **Matrix** | **Transcription Factor** | **Association Score** | ***P*-Value** |
| --- | --- | --- | --- | --- |
| 1 | NFKAPPAB_01 | Rela | 8.566 | 0.00E+00 |
| 2 | CREL_01 | C-rel | 8.287 | 0.00E+00 |
| 3 | NFKB_Q6_01 | Nf-kappab1, Nf-kappab2 | 7.065 | 0.00E+00 |
| 4 | NFKAPPAB65_01 | Rela | 7.061 | 0.00E+00 |
| 5 | NFKAPPAB50_01 | N/A | 6.186 | 0.00E+00 |
| 6 | STAT1_03 | Stat1alpha | 5.924 | 8.00E-06 |
| 7 | NFKB_C | N/A | 4.678 | 1.75E-04 |
| 8 | NFAT_Q4_01 | Nf-at1, Nf-at2 | 4.658 | 1.90E-04 |
| 9 | NFKB_Q6 | N/A | 4.630 | 2.06E-04 |
| 10 | STAT4_01 | Stat4 | 3.307 | 2.88E-03 |
| 11 | STAT6_02 | Stat6 | 3.225 | 3.73E-03 |
| 12 | TFE_Q6 | Mitf , Tfe3-l | 3.095 | 4.63E-03 |
| 13 | STAT3_02 | Stat3 | 3.094 | 4.63E-03 |
| 14 | LXR_Q3 | Lxr-alpha, Lxr-beta | 3.080 | 4.69E-03 |
| 15 | BACH2_01 | Bach2 | 2.941 | 7.05E-03 |
| 16 | STAT5A_03 | Stat5a | 2.928 | 7.24E-03 |
| 17 | BRACH_01 | Brachyury | 2.741 | 9.59E-03 |
| 18 | ATF6_01 | Atf6 | 2.718 | 1.17E-02 |
| 19 | HMGIY_Q6 | Hmg, Hmg-y | 2.578 | 1.38E-02 |
| 20 | ZID_01 | Zid | 2.548 | 1.64E-02 |
| 21 | EBOX_Q6_01 | Alf1a, Bhlhb2 | 2.525 | 1.68E-02 |
| 22 | E2F_Q4 | N/A | 2.498 | 1.70E-02 |
| 23 | USF_Q6 | Usf1, Usf2a | 2.451 | 1.84E-02 |
| 24 | GEN_INI_B | N/A | 2.388 | 2.23E-02 |
| 25 | STAT3_01 | Stat3 | 2.354 | 2.29E-02 |
| 26 | MYOGNF1_01 | Nf-1, Nf-1/l | 2.348 | 2.29E-02 |
| 27 | CDPCR3HD_01 | Cutl1 | 2.275 | 2.68E-02 |
| 28 | SREBP1_01 | Srebp-1, Srebp-1a | 2.275 | 2.68E-02 |
| 29 | USF2_Q6 | Usf2a | 2.275 | 2.68E-02 |
| 30 | MTF1_Q4 | Mtf-1 | 2.274 | 2.68E-02 |
| 31 | TBX5_Q5 | Tbx5, Tbx5 | 2.266 | 2.71E-02 |
| 32 | STAT1_01 | Stat1alpha, Stat1beta | 2.207 | 3.10E-02 |
| 33 | PBX1_01 | Pbx1a | 2.192 | 3.10E-02 |
| 34 | GEN_INI2_B | N/A | 2.176 | 3.38E-02 |
| 35 | PAX4_01 | Pax-4a | 2.150 | 3.48E-02 |
| 36 | AP1_01 | Fosb, Fra-1 | 2.150 | 3.48E-02 |
| 37 | AP1_Q6 | N/A | 2.150 | 3.48E-02 |
| 38 | USF_01 | Usf1 | 2.150 | 3.48E-02 |
| 39 | MZF1_01 | Mzf-1 | 2.120 | 3.91E-02 |
| 40 | AP1_Q4 | N/A | 2.063 | 4.19E-02 |
| 41 | AML_Q6 | Aml1, Aml1a | 2.054 | 4.22E-02 |
| 42 | PEBP_Q6 | Aml1, Aml1a | 2.054 | 4.22E-02 |
| 43 | STAT_01 | Stat1alpha, Stat1beta | 2.054 | 4.22E-02 |
| 44 | STRA13_01 | Stra13 | 2.053 | 4.22E-02 |
| 45 | USF_C | Usf1 | 2.053 | 4.22E-02 |
| 46 | LXR_DR4_Q3 | N/A | 1.992 | 4.82E-02 |
| 47 | TAACC_B | N/A | 1.975 | 4.93E-02 |
| 48 | TBX5_01 | Tbx5 | 1.975 | 4.93E-02 |
| 49 | AP1_Q2_01 | Fosb, Fra-1 | 1.971 | 4.99E-02 |
| 50 | MYCMAX_03 | Max, C-myc | 1.966 | 4.99E-02 |
