# Supplementary material for: Impact of Interleukin 10 Deficiency on Intestinal Epithelium Responses to Inflammatory Signals
Source: Front Immunol. 2021 Jun 16;12:690817. doi: 10.3389/fimmu.2021.690817 (PMC8244292; doi:10.3389/fimmu.2021.690817)
Supplement: Supplementary Figure 1 — Tagmentation fragments and qPCR analysis. [file DataSheet_1.docx]

**SUPPLEMENTARY INFORMATION**

**Figure S1. Tagmentation fragments and qPCR analysis.** Untreated enteroid cultures were used for nuclei purification, lysis and tagmentation reaction for 10 min. **(A)** The purified tagmented DNA was amplified by PCR and 1μL was used on Bioanalyser for analysis of DNA fragment sizes. **(B)** 2μL of the purified tagmented DNA was amplified by PCR reaction (volume = 50μl) for 5 cycles. 5μL of the partially amplified DNA was used as a template in a new qPCR reaction that included 1X SYBR green, in a total volume of 15μL. The amplification graphs (N=5) showed that 10 more cycles were enough to synthesize the DNA libraries without reaching plateau phase.

**
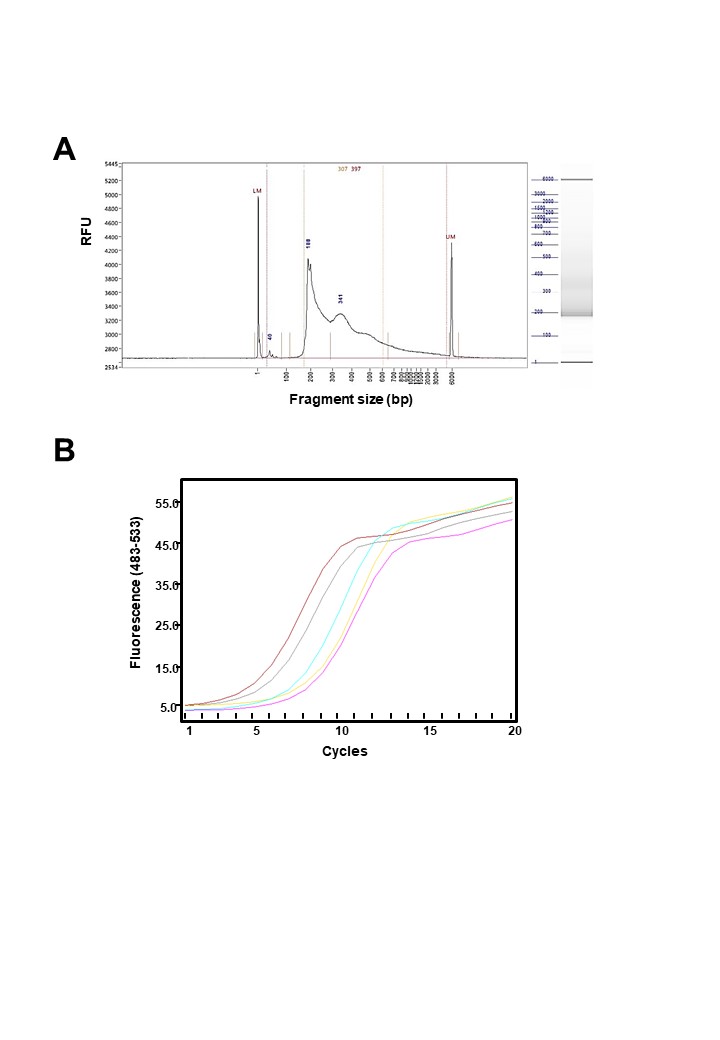
**
