# Supplementary material for: Impact of Interleukin 10 Deficiency on Intestinal Epithelium Responses to Inflammatory Signals
Source: Front Immunol. 2021 Jun 16;12:690817. doi: 10.3389/fimmu.2021.690817 (PMC8244292; doi:10.3389/fimmu.2021.690817)
Supplement: Supplementary Figure 2 — Proportion of TNF-induced genes identified as being common to both ATAC and RNA sequencing data sets. [file DataSheet_2.docx]

**SUPPLEMENTARY INFORMATION**

**Figure S2**. **Proportion of TNF-induced genes identified as being common to both RNA sequencing and ATAC sequencing data sets.** In enteroids treated for 2h with 40 ng/mL TNF, a comparison was made between genes identified as significantly upregulated (± 2-fold) as observed by RNA sequencing (n=296) and ATAC sequencing genes with annotated peaks within the promotor region up to 3 kb upstream of the transcription start site. **(A)** Venn diagram illustrating comparison made using all peaks identified by ATACseq in the TNF-stimulated sample combined with those peaks that were identified in the unstimulated sample as existing open chromatin sites (n=12,446 genes, from 22,016 annotated peaks); and **(B)** gene identities of the 216 genes common to both sequencing sets, with those in bold being known NFκB target genes. **(C)** Venn diagram illustrating comparison made using peaks identified by ATACseq that were unique only to TNF stimulated sample (n=688 genes, from 709 annotated peaks); **(D)** gene identities of the 25 genes common to both sequencing sets, with those in bold being known NFκB target genes. Venn diagram software freely available from Ghent University, at <http://bioinformatics.psb.ugent.be/webtools/Venn/> .

**A**


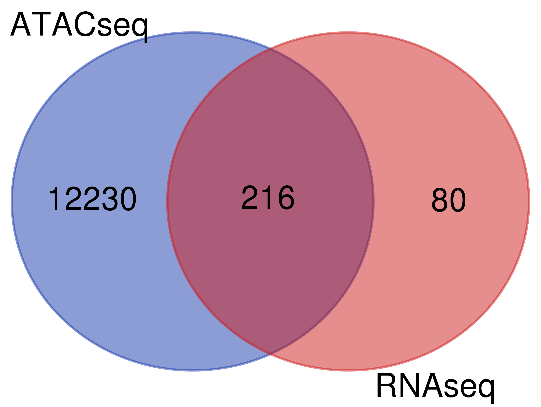


**B**

| **Names** | **Total** | **Genes** |
| --- | --- | --- |
| ATACseq RNAseq | 216 | *Katnbl1, Neil2, Baz1a, Dab2ip,* ***Fas****, Ppp4r1, Dok3, Gga3, Slc25a25, Rab22a, Arl5b, Urb1, Cacna1h, Chst8,* ***Tnfaip8****, Nploc4, Midn, Supt6, Prpf39, Rexo1, Sh3pxd2a, Gsdma2, Brd1, Hacd2,* ***Nfkb2****, Entpd7 Cspp1,* ***Dcp1a****, Taf1a, Kdm2a, Tfcp2l1, Tmem170b, Ints6, Etv3, Fbxw11, Chka, Syvn1, Pfkfb3, Vav2, Nup35,* ***Irf1****,* ***Ccl20****, Usp43, 1810013L24Rik, Psmd3,* ***Tnfaip3****,* ***Cxcl10****,* ***Hivep1****, Specc1, 9330151L19Rik, H2-K2, Brf1, H2-Q4, Cnnm4,* ***Nab1****, Adrm1, Trim39,* ***Myo10****,* ***Nos2****, Zgpat,* ***Icam1****, Morc3, Zfp429, Mybl2, Cdk12, Adat3, Malt1,* ***Nfkbiz****, Spata2, Camsap1, Yaf2,* ***Tlr2****,* ***Mafg****, Il6st,* ***C3****,* ***Ttyh2****, Necab3,* ***Rela****, Prrc2b Map3k9 Sbno2, Zfp335, Lama5, Spag1,* ***Yy1****, Senp5, Lrch3, Txnrd1, Sf1, Arhgap23, Ccnt1, Prdm4, Fam189a2, Vars, Jup, Tnfrsf11a, Clk4,* ***Tnip1****,* ***Dnase1l2****, Zswim6, Lin9,* ***Slk****, Cfap43, Pcnx3, Hells, Atg16l1, Rnps1, Itgb4,* ***Dido1****, Ranbp3, Srebf2, Cdk5r1, Col11a2, Ppp4r3b, D030056L22Rik,* ***Tlk1****, Kremen1, Trpv3, Jmjd6, Rhbdf1, Cep170b, Tmem39a, Cdc42bpb, Rnf31, Hist1h2bp, H2-Q5,* ***Nfkbia****, Tnks1bp1, Rptor, Tjap1, Hmgxb3, Nup153, Eif6,* ***Agps****, Hes1,* ***Ptger4****, Cluh, Atxn7, Neurl3, Rhbdl3, Zc3h13, Mroh2a, Sp5, Per2, Slmap, Zfp672, Clk1, Plekha6, Tcerg1, Srek1,* ***Traf2****,* ***Ripk1****,* ***Phlda1****, Csnk1d,* ***Tnf****, Sec61a2, Plec,* ***Bcl6****, Fbxo28, Ranbp9, Myo15b, Fam83g, Itgb6, Rhbdf2, Med16,* ***Chd1****, Prrc2a, Scrib, Donson, Idi1, Bptf,* ***Hmgcr****, Pgs1, Rcan1, Maff, Cxxc1,* ***Traf3****,* ***Hs6st1****, Pkmyt1,* ***Stk17b****,* ***Ahr****, Zbtb11,* ***Rel****,* ***Notch1****,* ***Stx11****, Tsc22d2, Akt1, Cactin, Safb,* ***Bmp2****, Cry1, Zfp207, Cabin1,* ***Csf1****, Hsd17b7, Psmd1, Fbxo33, Fam43a, Arid1b, Aoc3, Ctdp1, Fgfr1op, Spdl1, Stab1, Socs1, Tbk1, Rab32, Dnase1l3, Sik1, Elmsan1,* ***Foxo3****, Safb2,* ***Cxcl16****, Ern1, Incenp, Snx11* |

**C**

**
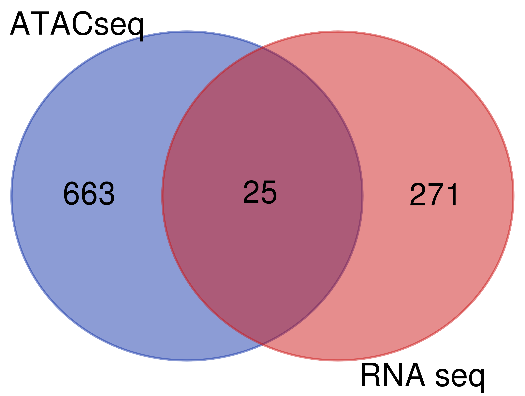
**

**D**

| **Names** | **Total** | **Genes** |
| --- | --- | --- |
| ATACseq RNAseq | 25 | *Nploc4, Plekha6, Rexo1, Sh3pxd2a,* ***Nos2****, Vars, Bptf, Dab2ip, Jup, Vav2,* ***Traf2****, Necab3, Prrc2b, Morc3,* ***Tnip1****,* ***Tnf****, Rptor, Slc25a25, Zfp672, Mybl2, Aoc3,* ***Notch1****, Cdc42bpb, Psmd3,* ***Tnfaip3*** |
