# Supplementary material for: Impact of Interleukin 10 Deficiency on Intestinal Epithelium Responses to Inflammatory Signals
Source: Front Immunol. 2021 Jun 16;12:690817. doi: 10.3389/fimmu.2021.690817 (PMC8244292; doi:10.3389/fimmu.2021.690817)

**SUPPLEMENTARY INFORMATION**

**Figure S3**: **Proportion of genes identified as NF**κ**B target genes from TNF-stimulated enteroid RNA sequencing.** All significantly altered genes observed in enteroids in response to 40 ng/mL TNF were compared to databases and publications reporting NFκB target genes. Charts demonstrate proportion of NFκB targets genes identified relative to the total number of significant gene changes at each timepoint, **(A)** 0.5h, **(B)** 1h, **(C)** 2h and **(D)** 24h post-TNF application: Red-blue represents increased-decreased gene expression compared to unstimulated controls. **(E)** Venn diagram showing 6 genes common to all time points in response to TNF treatment. Software used from Ghent University, freely available at http://bioinformatics.psb.ugent.be/webtools/Venn/


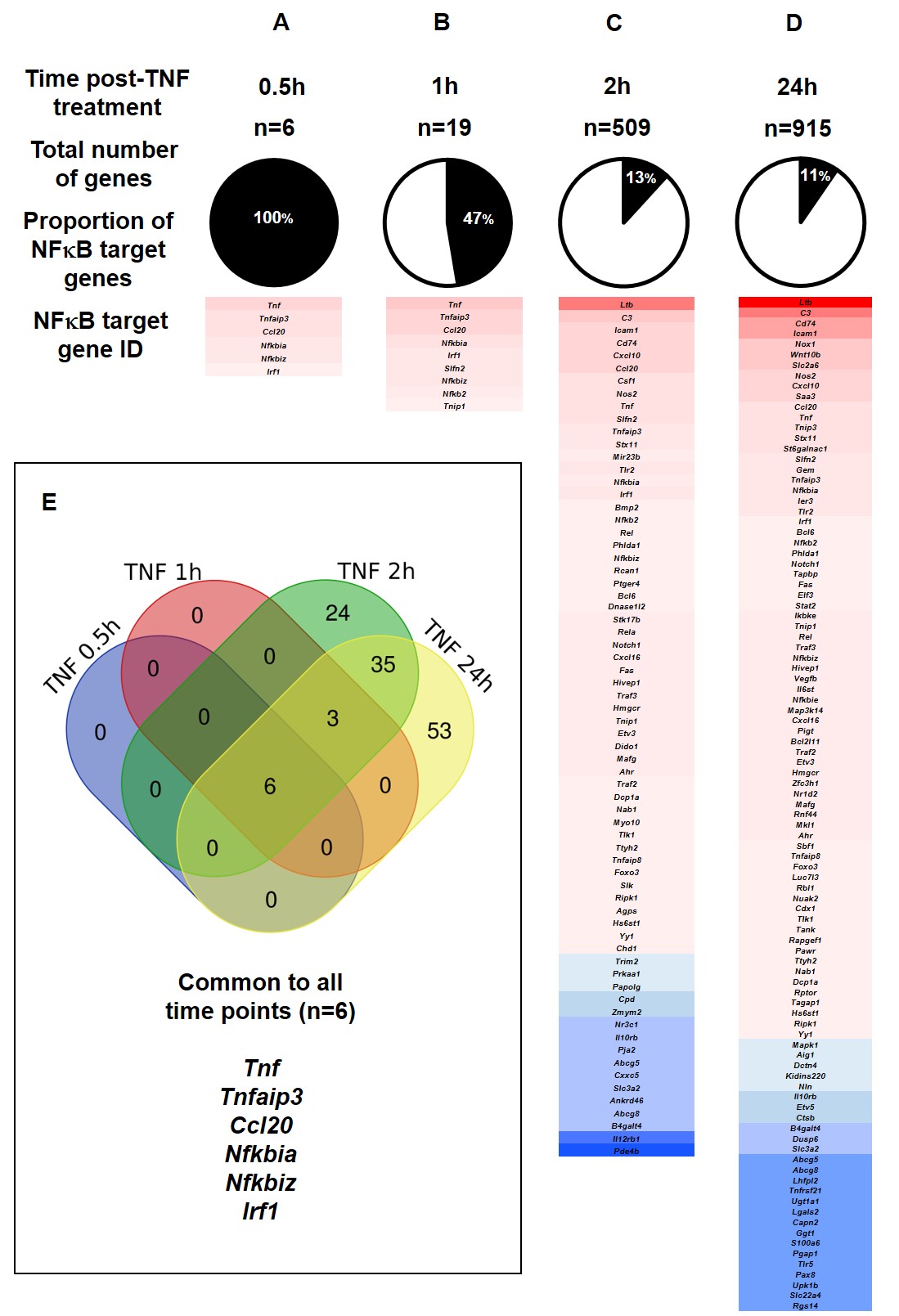

Supplement: Supplementary Figure 3 — Proportion of genes identified as NFkB target genes from TNF-stimulated enteroid RNA sequencing. [file DataSheet_3.docx]
