# Supplementary material for: Impact of Interleukin 10 Deficiency on Intestinal Epithelium Responses to Inflammatory Signals
Source: Front Immunol. 2021 Jun 16;12:690817. doi: 10.3389/fimmu.2021.690817 (PMC8244292; doi:10.3389/fimmu.2021.690817)

**SUPPLEMENTARY INFORMATION**

**Figure S4**: **qPCR analysis of early response NF**κ**B regulated genes in C57BL/6J enteroids**. **(A)** From the hierarchical cluster analysis five key NFκB regulated genes upregulated in response to TNF were identified within Cluster 7. Subsequent qPCR validation for these five target genes was performed on additional enteroid cultures, either left unstimulated or stimulated with **(B)** 40 ng/mL TNF, or **(C)** 100 ng/mL flagellin, for 1h. Data is presented as mean ± SEM (standard error of the mean); N=3-4 mice.


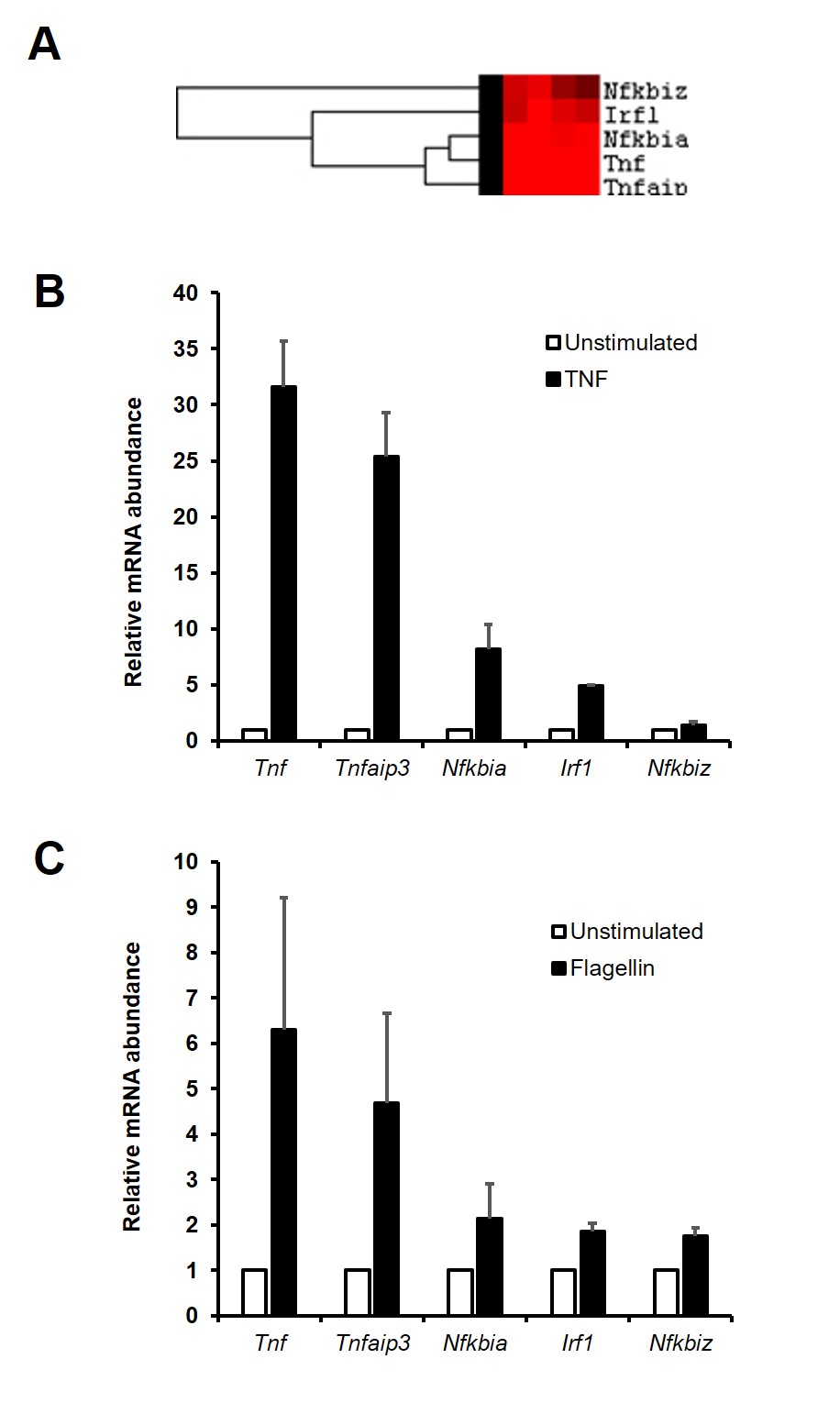

Supplement: Supplementary Figure 4 — qPCR analysis of early response NFkB regulated genes in C57BL/6J enteroids. [file DataSheet_4.docx]
