# Supplementary material for: Impact of Interleukin 10 Deficiency on Intestinal Epithelium Responses to Inflammatory Signals
Source: Front Immunol. 2021 Jun 16;12:690817. doi: 10.3389/fimmu.2021.690817 (PMC8244292; doi:10.3389/fimmu.2021.690817)
Supplement: Supplementary Figure 5 — Resting levels of NFkB target genes in enteroids deficient in interleukin 10. [file DataSheet_5.docx]

**SUPPLEMENTARY INFORMATION**

**Figure S5:** **NF**κ**B target genes encoding ABIN1 and ABIN2 are overexpressed in interleukin 10 deficient** **enteroids. (A)** Resting mRNA levels of *Tnip1* and *Tnip2* in *Il10^-/-^* enteroids as assessed by qPCR. Data expressed as fold change to levels in C57BL/6J cultures, with significant differences observed, **p*<0.05, Kruskal-Wallis test (N=3 mice). Dynamic gene expression in C57BL/6J and *Il10^-/-^* enteroids in response to stimulation over 8h with either **(B)** 40 ng/mL TNF, or **(C)** 100 ng/mL flagellin, compared to unstimulated controls. Data is presented as mean ± SEM (N=3 mice).

**
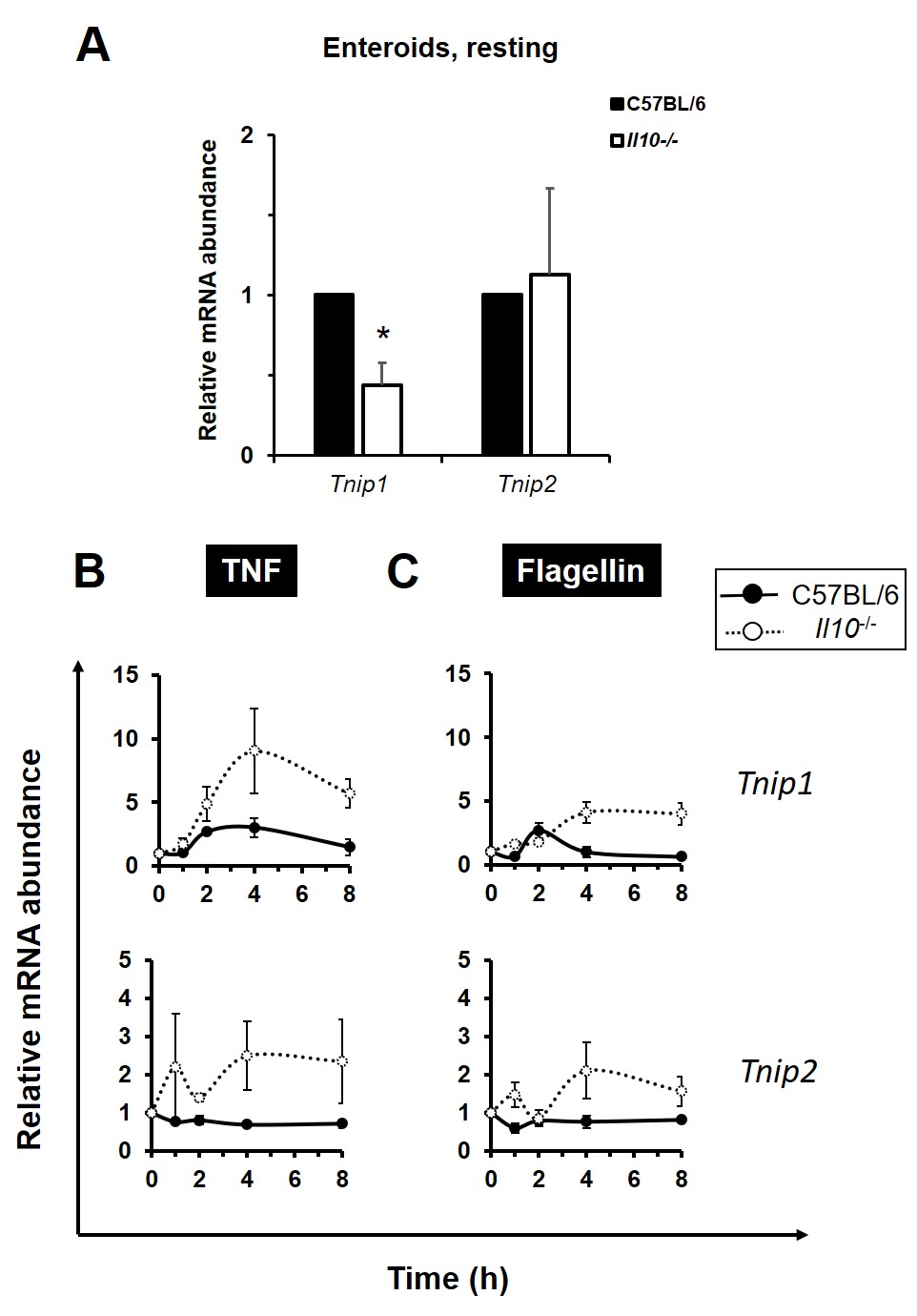
**
