# Supplementary material for: Impact of Interleukin 10 Deficiency on Intestinal Epithelium Responses to Inflammatory Signals
Source: Front Immunol. 2021 Jun 16;12:690817. doi: 10.3389/fimmu.2021.690817 (PMC8244292; doi:10.3389/fimmu.2021.690817)

**SUPPLEMENTARY INFORMATION**

**Figure S6**. **Resting levels of NF**κ**B target genes in enteroids deficient in interleukin 10.** Relative mRNA abundance of NFκB target genes in resting enteroid cultures from *Il10*^-/-^ mice expressed as fold change to levels observed in C57BL/6J wild-type controls. Significant differences compared to C57BL/6J, **p*<0.05 (Kruskal-Wallis test); N=3-4 mice.


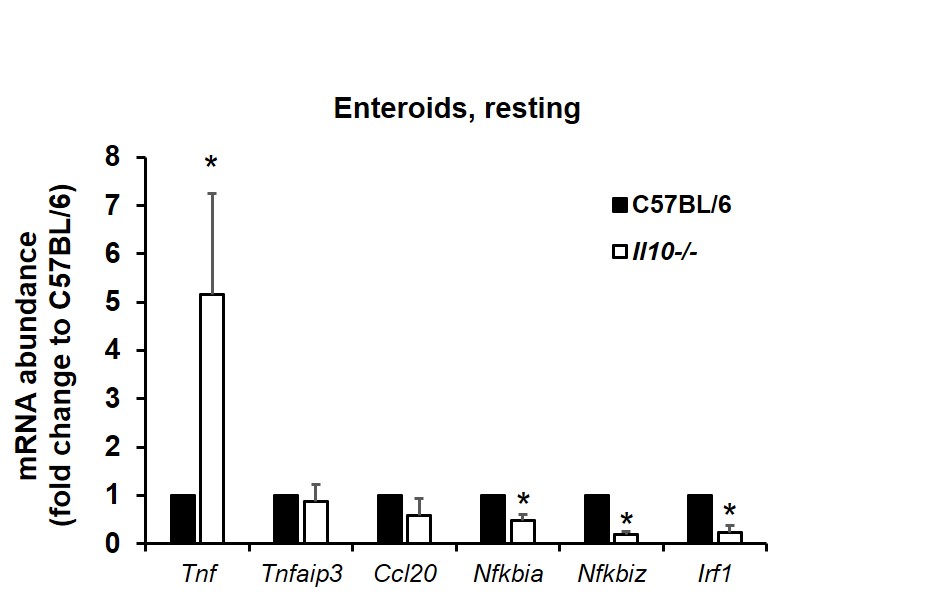

Supplement: Supplementary Figure 6 — NFkB target genes encoding ABIN1 and ABIN2 are overexpressed in interleukin 10 deficient enteroids. [file DataSheet_6.docx]
